# Thiol‐Based Neuroprotective Copolymers Acutely Restore Redox Metabolism and Mediate Vasogenic Edema in a Mouse Model of Traumatic Brain Injury

**DOI:** 10.1002/mabi.202500642

**Published:** 2026-07-21

**Authors:** Evan T. Curtis, Brandon Z. McDonald, Aria W. Tarudji, Aaron M. Priester, Anthony J. Convertine, Forrest M. Kievit

**Affiliations:** ^1^ Department of Biological Systems Engineering University of Nebraska‐Lincoln Lincoln Nebraska USA; ^2^ Department of Material Science and Engineering Missouri University of Science and Technology Rolla Missouri USA

**Keywords:** cellular metabolism, nanoparticles, oxidative stress, traumatic brain injury, vasogenic edema

## Abstract

Effective pharmaceutical interventions for treating the secondary damage associated with traumatic brain injury (TBI) are limited due to poor delivery into the brain, insufficient target engagement, and an incomplete understanding of the pathophysiological changes that occur post‐impact. Thus, nanoparticles (NP), which have an enhanced permeation and retention‐like effect within the perturbed blood‐brain barrier, have grown as a potential candidate for treating TBI. We have investigated the antioxidant capacity of thiol‐based NP, termed neuroprotective copolymers (NPC3), and their ability to neutralize reactive oxygen species (ROS) and lipid peroxidation products (LPOx). Here, we assessed the efficacy of NPC3 for alleviating the secondary injury cascade in TBI with a specific focus on ameliorating molecular and structural deficits in a mouse controlled cortical impact (CCI) model. NPC3 delivered post‐CCI alleviated oxidant burden, reducing both antioxidant enzyme expression and Nrf2 activation. These changes in redox signaling resulted in a shift in metabolic function, with increased AMPK activation with NPC3 treatment. T2‐weighted and diffusion magnetic resonance imaging revealed vasogenic edema formation at 30 days post‐CCI and alterations in mean diffusivity, which were moderated by NPC3. Furthermore, NPC3 reduced GFAP and Iba1 at multiple impact severities, which positively correlated with urinary 8‐isoprostane. Overall, this work shows NPC3 reduced glial reactivity, affected redox metabolism, and ultimately contributed to improvements in structural deficits post‐CCI.

## Graphical abstract

In this study, we assess the efficacy of an antioxidant nanoparticle system, NPC3, for alleviating the secondary injury cascade in TBI with a specific focus on ameliorating molecular and structural deficits in a mouse controlled cortical impact model. Delivering NPC3 treatment post‐injury alleviates oxidant burden, reducing both antioxidant enzyme expression and Nrf2 activation and moderates vasogenic edema.

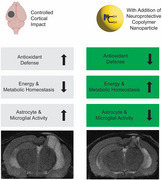

## 1 Introduction

Traumatic brain injury (TBI) is one of the most common neurological disorders, with incidence rates in New Zealand and North America between 811 and 979 per 100 000 people per year, and is caused by a jolt, bump, or blow to the head [1]. These primary injuries to the brain can be highly variable due to the multitude of primary injury sources such as road traffic incidents, falls, or sports related events [2]. TBI has a strong causal link to issues such as Chronic Traumatic Encephalopathy, Alzheimer's disease, Parkinson's disease, and epilepsy [3, 4, 5, 6]. Furthermore, interventions to address the secondary complications of TBI are limited, in part, because of poor delivery, limited target engagement, and an incomplete understanding of the pathophysiological changes that occur post‐impact [7, 8, 9, 10].

Following the primary impact, there is a cascade of events that can result in secondary expansion and progression of the injury. Critical drivers of secondary injury are reactive oxygen species (ROS) and lipid peroxidation products (LPOx). This oxidative stress environment leads to significant cell death, neuroinflammation, and neurodegeneration caused by a complex interaction of cellular components, subcellular components, and immune responses [11]. Most prominent in severe TBI is the formation of a glial scar to wall off post‐traumatic necrosis that gives way to an edematous lesion [12]. Thus, a major goal in TBI treatment has been to reduce post‐traumatic oxidative stress in hopes of reducing the secondary injury cascade and preserving cortical tissue by limiting necrosis and neuroinflammation.

Nanoparticles (NP) offer one strategy to improve brain delivery and target engagement because of an enhanced permeation and retention‐like effect in severe TBI [13, 14]. The breakdown of the BBB allows NP to passively accumulate in the injured brain and increase exposure due to their larger size compared to small molecule drugs that prevents their rapid diffusion out of the injury [13, 14, 15, 16]. Indeed, we found that antioxidant NPs accumulate in the damaged brain and exert a therapeutic effect [17, 18, 19, 20].

We recently developed neuroprotective copolymer nanoparticles (NPC3) that contain thiol groups capable of inactivating both ROS and LPOx to limit the negative effects of these molecules and improve behavioral outcomes [21, 22]. However, the time course of how the treatment affects structural and molecular changes is still unknown. These data could help better our understanding of antioxidant NP treatment effects during injury progression. Therefore, we investigated the effect of NPC3 in a severe controlled cortical impact (CCI) model of TBI through evaluation of diffusion magnetic resonance imaging (MRI), immunoblotting, and confocal imaging.

## 2 Materials and Methods

### 2.1 NPC3 Synthesis and Characterization

NPC3 synthesis was conducted as previously described [21, 22]. Poly(ethylene glycol) methyl ether methacrylate monomer (O950; 4.00 g, 4.21 mmol), lipoic acid methacrylate monomer (LIPOMA; 1.00 g, 3.10 mmol), 3.59 mL of a 25 mg mL^−1^ of 4‐((((2‐carboxyethyl)thio)carbonothioyl)thio)−4‐cyanopentanoic acid (CCC) stock in dimethylacetamide (DMAc; 90 mg, 0.293 mmol), 410 µL of a 10 mg mL^−1^ 4,4′ azobis (4‐cyanovaleric acid) (ABCVA) in dimethyl sulfoxide (DMSO) (4.1 mg, 0.015 mmol), DMAc (3.5 g), and 1.15 g of sodium borohydride (NaBH_4_; 31 mmol, 10:1 M ratio) were reacted under argon gas at 70°C for 24 h. NaBH_4_ is used to reduce NPC3 to an active form via generating free thiol functional groups. The polymers were dialyzed in a Spectrapor regenerated cellulose dialysis membrane against ethanol for one day followed by deionized (DI) water for two days, before they were freeze‐dried.

#### 2.1.1 NMR Procedure

^1^H NMR samples were prepared by dissolving unreduced NPC3 and reduced NPC3 deuterated chloroform (CDCl_3_) and deuterium oxide (D_2_O), respectively, to a concentration of 10 mg‐mL^−1^. 450 µL of each solution were injected into glass NMR tubes, and samples were run on a 400 MHz Bruker proton NMR. Following measurement, spectra were analyzed using Bruker TopSpin analysis software and data were plotted using GraphPad.

#### 2.1.2 GPC Procedure

Standards of poly(methyl methacrylate) (PMMA) were first dissolved in DMF + 1% LiBr at a concentration of 5 mg‐mL^−1^ and run on the Agilent Infinity II Gel Permeation Chromatographer (GPC) using a flow rate of 0.5 mL‐min^−1^. These GPC elution times and molecular weights for the standards were then used to construct a calibration curve. Next, unreduced and reduced NPC3 polymer samples were dissolved in DMF + 1% LiBr at the same concentration and measured using the same flow rate on the GPC. Once collected, the calibration curve was used to determine the molecular weight of the dissolved NPC3 polymers in solution along with a molar mass dispersity or polydispersity index (PDI). Data was extracted into Microsoft Excel and was plotted using GraphPad.

#### 2.1.3 Ellman's Reagent Assay for Reduced NPC3 Polymer

Molar concentrations of thiols per gram of polymer were determined using Ellman's DTNB assay. A standard curve was first constructed using L‐cysteine as the thiol source. A procedure for this is shown separately below. Stock of reduced copolymer (10 mg‐mL^−1^) in DI water and Ellman's reagent (3 mg‐mL^−1^) in pH 7 sodium phosphate buffer were first prepared. The reduced copolymer stock was used to create a series of sample concentrations by diluting 0, 10, 20, 30 or 40 µL of the stock into 3 mL of PBS buffer (100 mM, pH 7) followed by 50 µL addition of Ellman's reagent solution to each sample. After 15 min of incubation, 200 µL of each sample was pipetted into a 96‐welll plate, and absorbance was measured at 412 nm. Using absolute absorbance values and the standard curve, moles of thiols per gram of polymer were calculated and compared to theoretical values.

#### 2.1.4 Standard Curve for Ellman's Reagent Assay

L‐cysteine stock solution in DI water (3 mM) was used to prepare a range of sample concentrations (0 to 40 µM) by diluting 0 – 40 µL of L‐cysteine stock in PBS (100 mM, pH 7). To each sample, 50 µL of Ellman's solution (3 mg‐mL^−1^ in pH 7 PBS) was added. Samples (200 µL) were then transferred to a BD Falcon 96‐well clear flat bottom plates. After 15 min, absorbance was measured at 412 nm on the TECAN Infinite M Nano+ Plate Well reader. Absolute absorbance values (cm^−1^) were plotted versus concentration to obtain a linear plot (R^2^ = 1) and molar absorptivity coefficient (ε, 14170 (M‐cm)^−1^), which was in good agreement with literature (ε, 14150 (M‐cm)^−1^) [23].

### 2.2 Controlled Cortical Impact and Drug Administration

All experimental surgeries were approved by the Animal Care and Use Committee at the University of Nebraska — Lincoln (UNL IACUC approval number 2300). Injuries were performed on 8‐week‐old male C57BL/6J mice (Jackson Laboratory, Bar Harbor, ME) utilizing an electromagnetic controlled cortical impact (CCI) device (PCI3000, Hatteras Instruments), as described previously [19, 20, 22, 24]. All mice were randomized to one of three groups, including naïve (CTRL), CCI, and CCI+NPC3, such that each cage contained each treatment group. Briefly, animals were anesthetized and positioned into a stereotaxic frame (Model 963, David Kopf Instruments), where anesthesia was maintained at 2.0%. Mice received dose sustained‐release buprenorphine (0.5 mg/mL, 60 µL) via dorsal subcutaneous injection and a topical anesthetic, following hair removal, containing lidocaine (5 mg/mL) and bupivacaine (0.3 mg/mL). A midline incision (1 cm) was made to reveal the skull after antiseptic treatment via iodine, followed by a 2.7 mm craniectomy at 2 mm posterior to bregma and 2 mm left of midline. The CCI device delivered a stainless steel impactor (2 mm diameter) at a velocity and dwell time of 4 m/s and 80 ms, respectively, with injury severity varied by an impact depth of 1.5 or 2.5 mm. Immediately post‐CCI, mice designated to the CCI+NPC3 group were treated with a single dose of NPC3 (8 mg/kg) via intravenous (IV) administration. The delivery time point and selected dose of 8 mg/kg were determined based on our previous treatment window and dose‐response studies [22]. All NPC3 was delivered in the reduced form as NPC3's therapeutic efficacy necessitates the formation of free thiol functional groups. Mice in the control and CCI only groups did not receive NPC3 treatment.

### 2.3 Antibodies

Tables 1 and 2 provide a list of primary and secondary antibodies used for immunoblotting (IB) and immunohistochemistry (IHC). (Tables 1 and 2).

**TABLE 1:** Primary and secondary antibodies used for immunoblotting (IB).

Primary antibody	Host species	Antibody dilution	Company, Catalog No.
β‐Actin	Mouse	1:2000	Sigma–Aldrich, A2228
GFAP	Rabbit	1:1000	Abcam, ab7260
Catalase	Rabbit	1:1000	Abcam, ab52477
Peroxiredoxin 6	Rabbit	1:1000	Abcam, ab133348
Peroxiredoxin 5	Rabbit	1:1000	Abcam, ab180587
Glutathione Peroxidase 1	Rabbit	1:1000	Abcam, ab22604
Heme Oxygenase 1	Rabbit	1:1000	Abcam, ab13243
Phospho mTOR	Rabbit	1:500	Cell Signaling, 2971S
Phospho AMPK	Rabbit	1:500	Cell Signaling, 2531S

Primary antibody	Host species	Antibody dilution	Company, Catalog No.
Anti‐Rabbit	Goat	1:10 000	BioRad, 1705046
Anti‐Mouse	Goat	1:10 000	BioRad, 1705047

**TABLE 2:** Primary and secondary antibodies used for Immunohistochemistry (IHC).

Primary antibody	Host species	Antibody dilution	Company, Catalog No.
GFAP	Goat	1:250	Abcam, ab53554
Iba1	Rabbit	1:1,000	Wako, 019–19741

Secondary antibody	Host species	Antibody dilution	Company, Catalog No.
Anti‐Rabbit	Donkey	1:250	Abcam, ab150074
Anti‐Goat	Donkey	1:250	Abcam, ab150129

### 2.4 In Vivo MRI Image Acquisition

Mice were imaged on a 9.4T MRI system (Varian, Palo Alto, CA) prior to and 1, 3, 7, and 30 days following surgery. The MRI system was equipped with a 4 cm Millipede RF imaging probe with triple‐axis gradients (100 G/cm max, 1000 mT/m). The software used for imaging was VnmrJ 3.0c (Agilent, Santa Clara, CA). All animals were anesthetized with 1.5%–2.0% isoflurane during scanning. Breathing was monitored and maintained between 50–80 breaths per minute (Small Animal Instruments, inc., Stony Brook, NY). For in vivo structural imaging, images were acquired with a fast spin‐echo multi‐slice sequence (FSEMS)—Repetition time (TR) = 5500 ms, echo spacing (ESP) = 12.5 ms, echo train length/ number of segments (ETL/ Seg) = 64/4, k‐zero = 4, effective echo time (effTE) = 50.02 ms, Averages = 2, Matrix size = 256 × 256, field of view (FOV) = 20 mm × 20 mm, slice thickness = 1 mm, number of slices = 15, effective in‐plane resolution = 78 µm × 78 µm (scan time = 11 min 55 s). Diffusion acquisitions were acquired with a 2D spin‐echo multi‐slice with diffusion tensor imaging sequence (2D SEMS‐DTI)—TR = 2000 ms, TE = 22.98 ms, Averages = 1, Matrix size = 128 × 128, effective in‐plane resolution = 156 µm × 156 µm diffusion parameters: Jones6 (1 b = 0 directions, 6 direction sensitizations), amplitude = 19.04 G/cm, duration = 6.0 ms, separation = 12.0 ms, b‐value 1000 s/ mm^2^ (scan time = 29 min 52 s).

### 2.5 Ex Vivo MRI Image Acquisition

A 3D spin‐echo sequence (3DSE), with diffusion parameter options selected, was applied to acquire diffusion weighted images of the ex vivo brain—TR 600 ms, TE 24 ms, Averages 1, Matrix 96 × 96 × 96, gradient spoil on, FOV 20 mm × 20 mm × 20 mm, diffusion parameters: Jones30 (1 b = 0 direction, 30 direction sensitizations), amplitude 33.1 G/cm, duration 6 ms, separation 12 ms, target b‐value = 3000 s/mm^2^ (scan time 47 h 37 min). For ex vivo MRI, a different cohort of mice was utilized because acquisition times for diffusion tractography assessments were substantially longer (∼47 h) compared to in vivo MRI (∼11 and 30 min), precluding imaging with the same animals.

### 2.6 MRI Processing

For T2‐weighted image analysis, regions of interest (ROI) were drawn by a blinded experimentalist using ITK‐SNAP (ver 3.6). Lesion regions were identified by hyperintense/hypointense signal relative to the surrounding tissue as described previously [25]. ITK‐SNAP calculates the volume and voxel values once the segmentation of the structure has been determined. The criteria for selecting the non‐anatomical regions was based on the assumption that lesioned tissue (i.e., brain region affected by TBI) displays a hyperintense signal relative to the surrounding tissue. DTI analysis used DSI Studio to generate FA and MD maps for each mouse. The data were fitted with the tensor model using DSI Studio (http://dsi‐studio.labsolver.org, ver. Chen 20230523) to calculate standard DTI metrics (fractional anisotropy FA, mean diffusivity MD). Sample size (N) represents biological replicates and the same sample size is used in each time point of the longitudinal study.

### 2.7 Lysate Preparation and Immunoblotting

Perfused brains were collected at 1, 3, and 7 days post‐CCI, separated into four regions including: ipsilateral and contralateral cortices and hippocampi, and homogenized in 300 µL of RIPA buffer (50 mM Tris HCl pH 8.0, 150 mM NaCl, 1% Triton X‐100, 0.5% Na Deoxycholate, 0.1% SDS, 1 mM EDTA, 0.5 mM EGTA, 1 mM PMSF, 1 mM Na3VO4, 1 mM NaF) using a TissueLyser II (Qiagen). The samples were horn‐sonicated (20s, 20% pulse), centrifuged (17,740 rcf, 4°C, 5 min), and a bicinchoninic acid (BCA) assay was used to determine supernatant protein concentration. Lysates were prepared with β‐Mercaptoethanol and 4x Laemmli Buffer (1:9), boiled (95°C, 5 min), and stored at −20°C. For immunoblotting, proteins were separated via SDS‐PAGE (120 V, 80 min) and transferred to PVDF membranes using a Trans‐Blot Turbo. After washing, membranes were blocked in 5% Blot‐Quick Blocker or BSA in TBST for 1 h at room temperature and incubated with primary antibodies at 4°C overnight. Membranes were washed in TBST, incubated with secondary antibody (1 h), and washed again before developing with ECL reagent (5 min) and imaging.

### 2.8 Immunoblotting Quantification

ROI were hand drawn around each respective band using the “freehand” volume tool in Image Lab (Version 6.0.1 BioRad) to obtain optical densitometry (OD) data. Using local background subtraction, adjusted volume intensity (AVI) values for target proteins were divided by AVI values from their respective loading control bands (i.e., B‐actin) to achieve normalized OD data. Normalized OD data for control samples from each blot were averaged and each normalized OD value was divided by this average to obtain a ratio to CTRL, which serves as the y‐axis for all immunoblotting data. All data were collected from appropriately exposed non‐saturated blots. Image Lab files and raw data are available in the Supporting Information. Sample size (N) represents biological replicates, where the same control samples were used for comparison against CCI and CCI+NPC3 groups.

### 2.9 Cryosection Preparation and Immunostaining

Mice were transcardially perfused with ice‐cold 4% PFA at 7 d post‐injury. Brain tissues were then collected and fixed in PFA overnight, followed by 30% sucrose for cryoprotection. Brains were embedded in OCT compound, coronally sliced with a 15 µm thickness, and laid on Epredia Polysine‐coated microscope slides (P4981001, Epredia). The sections were dried overnight at RT and stored at ‐80°C until use. Sections were washed with DPBS three times and blocked with blocking buffer (3% donkey serum, 0.3% Triton X‐100, and 0.1% sodium azide in DPBS) for 1 h at RT. The primary and secondary antibodies (Ab) were diluted in the blocking buffer. Sections were incubated with primary Ab against GFAP and Iba1 for 24 h at 4°C. Sections were washed with blocking buffer for three times for 5 min each, and incubated with secondary Ab of AF488 labeled donkey anti‐goat and AF555 labeled donkey anti‐rabbit for 2 h at RT. Sections were washed with the blocking buffer for three times for 5 min each, stained with DAPI for 5 min, rinsed with DPBS, rinsed with DDI water, and mounted with ProLong Gold Antifade Mountant (P36934, ThermoFisher Scientific). Images were acquired with Zeiss Confocal Microscope LSM 800 at 20x objective lens magnification.

### 2.10 Immunohistochemistry Quantification

GFAP and Iba1 were quantified from a single 20x field of view from each region of each mouse. The quantification of GFAP+ astrocytes and Iba1+ microglia was counted manually in ImageJ software. GFAP+ and Iba1+ cells were determined by colocalization between DAPI and GFAP or Iba1, respectively; GFAP and Iba1 without DAPI were omitted. GFAP+ and Iba1+ cells were then divided by the total area of the image field (mm^2^) to find the density (cells/mm^2^) in the regions. The proportional area measurement of GFAP and Iba1 (%area) was determined by the proportional area of tissue occupied by thresholded immunohistochemical staining using ImageJ software. The threshold was set above the autofluorescence background of control brain and kept the same for all of the samples. The average area of astrocyte and microglia (µm^2^/cell) was counted by multiplying the % area by the area of image field divided by the number of cells in the respective image. Sample size (N) represents biological replicates.

### 2.11 ELISA Kit Assay

Urinal free 8‐isoprostane concentration was determined with 8‐Isoprostane ELISA Kit (516351, Cayman Chemicals, USA) according to the manufacturer's instructions. The samples did not go through hydrolysis, thus only measured the free/unconjugated 8‐isoprostane in the urine. The concentration of 8‐isoprostane was normalized to the concentration of creatinine in the urine and expressed in nanogram per milligram creatinine. Sample size (N) represents biological replicates and the same sample size is used in each time point of the longitudinal study.

### 2.12 Creatinine

Creatinine concentration was determined with Creatinine (urinary) Colorimetric Assay Kit (500701, Cayman Chemicals, USA) according to the manufacturer's instruction. Sample size (N) represents biological replicates and the same sample size is used in each time point of the longitudinal study.

### 2.13 Statistical Analysis

All statistical analysis and graphs were generated using GraphPad Prism (version 10.1). Power analysis for immunoblotting and immunohistochemistry was performed to determine sample size for detecting statistical differences between CTRL and CCI based on our previous in vivo studies [19, 20, 22, 24]. No power analysis was conducted for MRI or ELISA studies. For immunoblotting and immunohistochemistry, all tests were conducted using a Two‐Way ANOVA with Tukey's multiple comparisons test for post‐hoc analysis. For MRI acquisition, two‐way ANOVA with Tukey's multiple comparisons test was completed for both lesion size and mean diffusivity, while ex vivo acquisition was completed using a One‐Way ANOVA and Welch's unpaired t‐test for number of tracts and lesion size, respectively.

## 3 Results

### 3.1 NPC3 Characterization and Experimental Timeline

**FIGURE 1:**
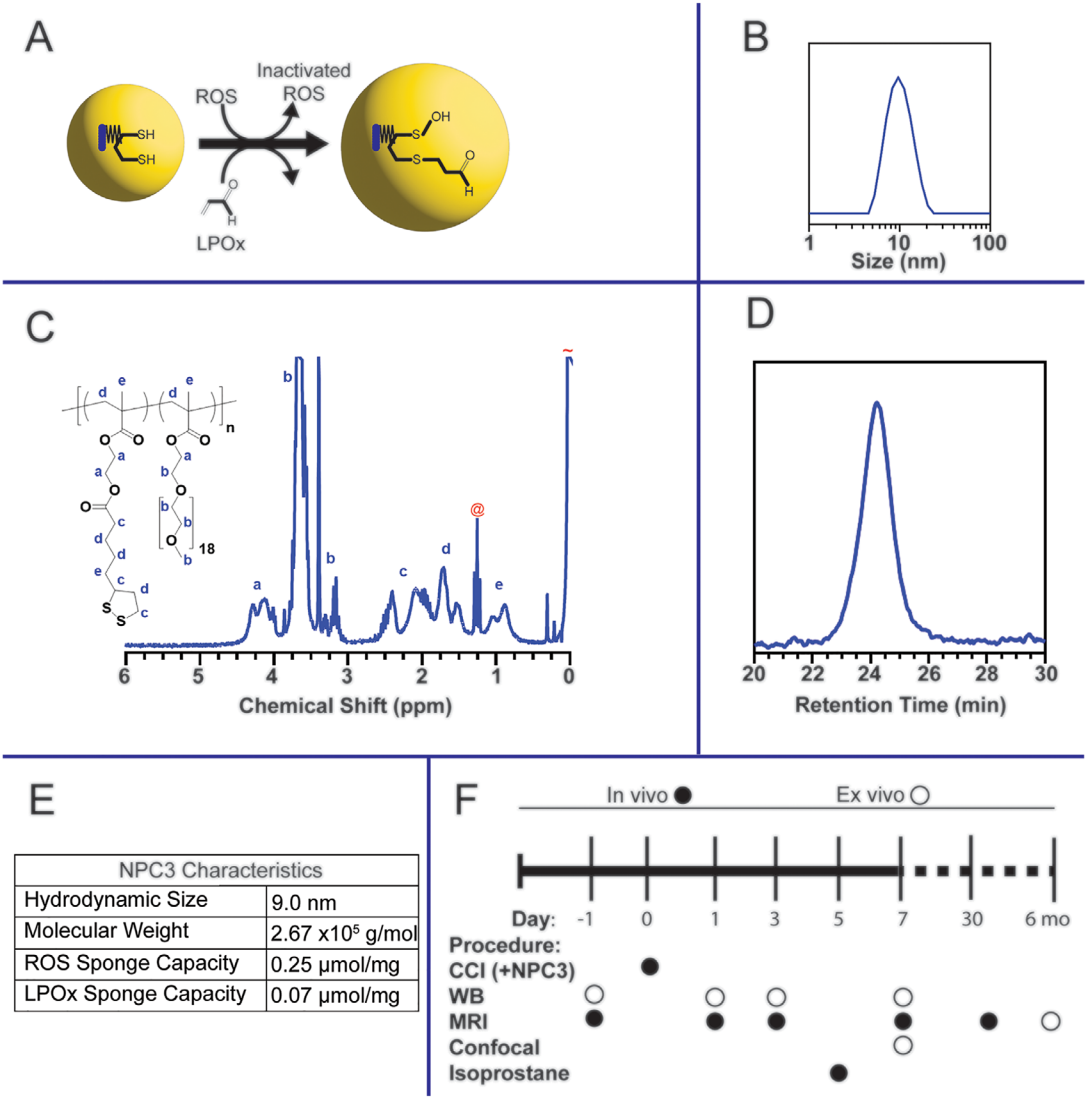
Schematic of NPC3, characterization, and experimental timeline. (A) ROS and LPOx scavenging reaction of NPC3. (B) DLS for reduced NPC3 in buffered H_2_O. (C) NMR purified, unreduced NPC3 in CDCl_3_ –*Note*: @ is residual ethanol from purification process; ∼ is TMS from chloroform–. (D) GPC trace for reduced NPC3 in DMF + 1% LiBr (flow rate 0.5 mg/min). E) Table summarizing the characteristics of NPC3. F) Timeline for experiments showing when either in vivo or ex vivo procedures took place.

The synthesis and characterization of NPC3 have been conducted previously and the same synthesized batch was utilized in the current study [21, 22]. NPC3 is a reversible addition‐fragmentation chain transfer (RAFT) polymer designed to scavenge ROS and LPOx (Figure 1A). Our previous analysis utilized dynamic light scattering to confirm that NPC3 had a hydrodynamic diameter of 9 nm (Figure 1B), while proton nuclear magnetic resonance (^1^ HNMR) verified the chemical structure of NPC3 (Figure 1B,C). Using gel permeation chromatography (GPC), we demonstrated that NPC3 has a retention time of 24 min and polydispersity index of 0.16 [21, 22], indicative of a relatively monodisperse distribution in solution (Figure 1D). The molecular weight of the polymer was determined to be 26 700 g/mol in close to the theoretical molecular weight of 17 400 g/mol, where differences are likely due to polymethyl methacrylate standards used for fabrication [21]. Following reduction and purification, the RAFT polymer was characterized for thiols using Ellman's reagent assay, yielding a value of 0.8 mmol SH/g of polymer, consistent with a scavenging capacity of 0.25 µmol ROS and 0.07 µmol LPOx per mg polymer (Figure 1E). ROS and LPOx sponge capacity were estimated from their in vitro efficacy using SH‐SY5Y neuroblastoma cells [21]. The overall therapeutic efficacy of NPC3 was assessed with a CCI mouse model based on the experimental timeline provided below (Figure 1F).

### 3.2 NPC3 Intervention Acutely Reduced GFAP Expression in the Ipsilateral Cortex Post‐CCI

**FIGURE 2:**
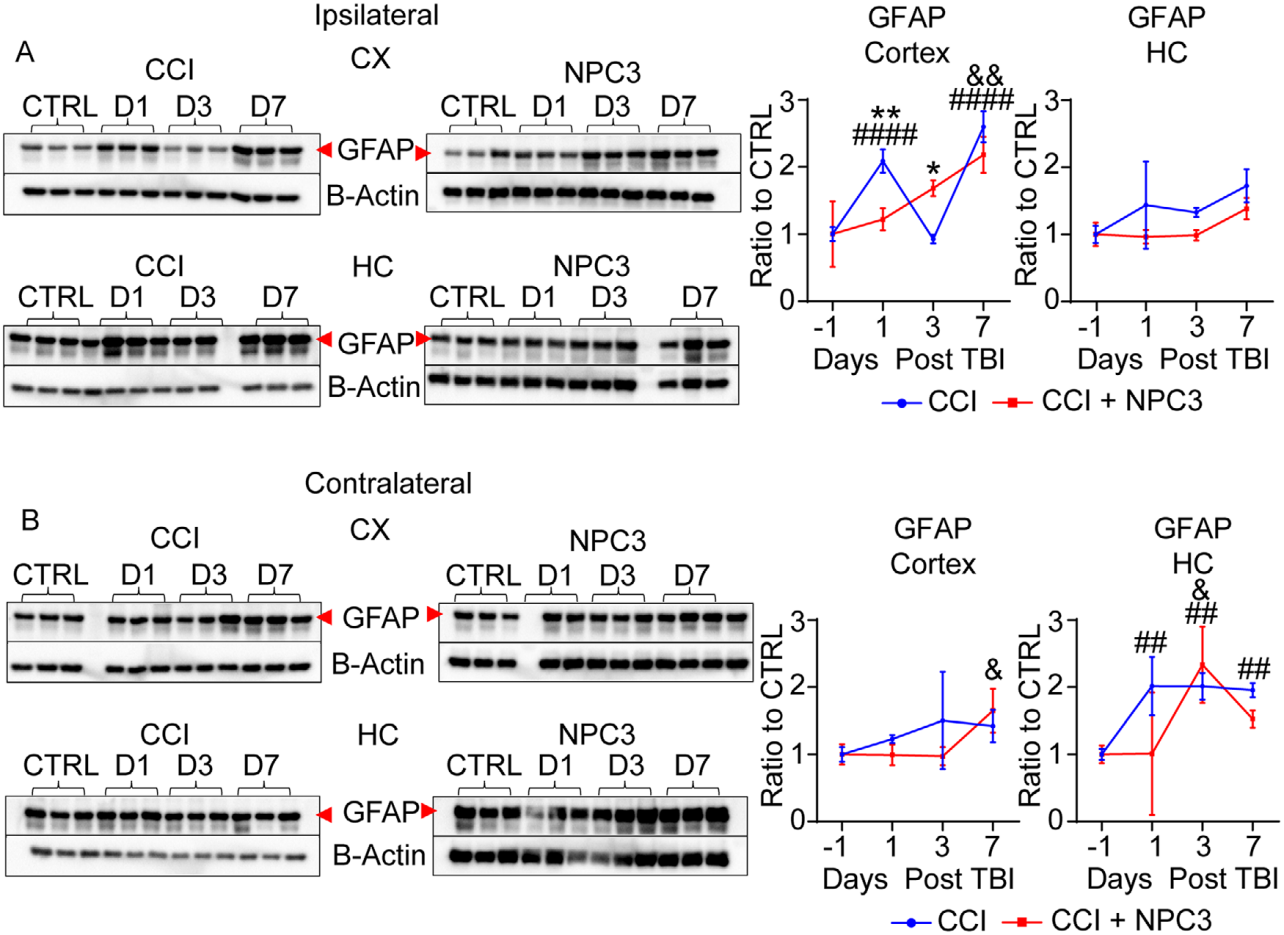
Effect of NPC3 on GFAP expression post 2.5 mm depth CCI. (A) Western blot images and data of GFAP from the ipsilateral cortex (CX) and hippocampus (HC). Red arrows are used to identify the top band in the GFAP panels used for quantification. GFAP expression was increased in the cortex at 1 and 7 days post‐CCI and significantly differed from CCI+NPC3 at 1 and 3 days post‐CCI. No significant differences were observed in the HC, although GFAP expression was lower at each time point following the treatment of NPC3. (B) Western blot images and data of GFAP from the contralateral CX and HC. GFAP expression increased in the HC at each time point post‐CCI. No significant differences between CCI and CCI+NPC3 were observed in either region, although expression levels were reduced at 1 day following NPC3 treatment. Data are represented as mean ± SD (N = 3/treatment/day; CTRL samples were used for both CCI and CCI+NPC3 blots; N = 21 total) #: CTRL vs CCI; and: CTRL vs CCI + NPC3; *:CCI vs CCI + NPC3 *:*p *<0.05; **:*p *<0.01; ***: *p *<0.001; ****: *p *<0.0001.

Astrocytes directly regulate the function and survival of neurons through nutritional, chemical, and structural support [26]. Additionally, astrocytes contribute to structural restoration and synaptic reorganization in TBI and expression changes provides valuable diagnostic information for examining the efficacy of potential therapeutic strategies [27]. We examined changes in the astrocytic marker, glial fibrillary acidic protein (GFAP), post‐CCI via immunoblotting. GFAP expression increased in the ipsilateral cortex at 1 and 7 days post‐CCI and was significantly reduced at 1 day following NPC3 treatment (Figure 2A). No significant differences were observed in the ipsilateral hippocampus, despite a trending lower expression at each time point. Additionally, GFAP expression was significantly increased in the contralateral hippocampus at each time post‐CCI (Figure 2B). All references to the ipsilateral and contralateral hippocampus throughout the manuscript are defined relative to the site of cortical injury.

### 3.3 NPC3 Intervention Reduces Antioxidant Defense Expression and Activity Post‐CCI

**FIGURE 3:**
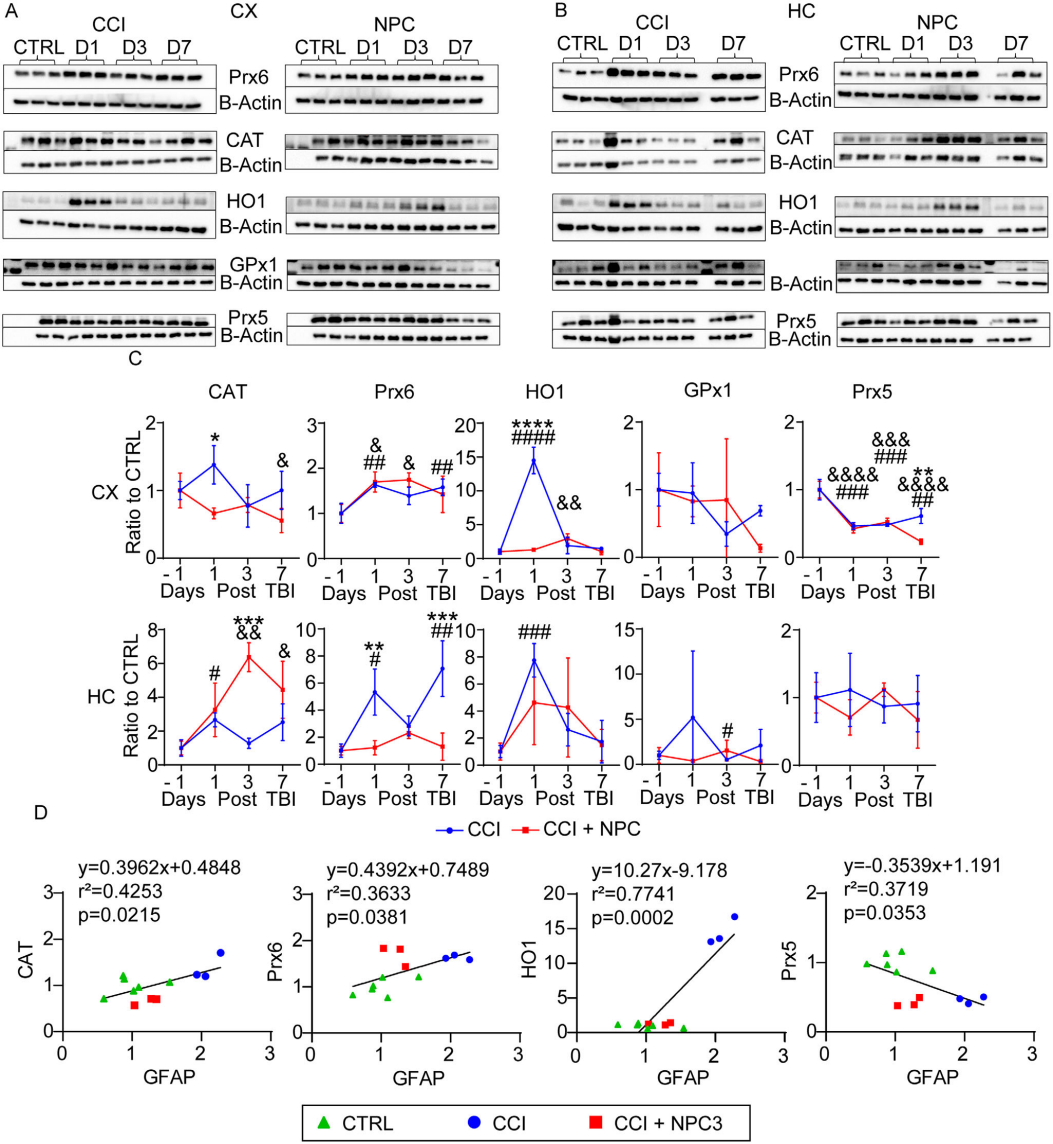
Effect of NPC3 on antioxidant defense post 2.5 mm depth CCI. Western blot images of markers of antioxidant defense from the (A) ipsilateral cortex (CX) and (B) hippocampus (HC). (C) Catalase (CAT) and heme oxygenase 1 (HO1) peaked at 1 day post‐CCI in the ipsilateral CX, which significantly differed from CCI+NPC3. Prx6 peaked at 1 and 7 days post‐CCI in the ipsilateral HC and significantly differed from CCI + NPC3. (D) CAT, Prx6, and HO1 positively correlated with GFAP expression at 1 day post‐CCI in the ipsilateral cortex, while Prx5 was negatively correlated. Data are represented as mean ± SD (N = 3/treatment/day; CTRL samples were used for both CCI and CCI+NPC3 blots; N = 21 total). #: CTRL vs CCI; &: CTRL vs CCI + NPC3; *:CCI vs CCI + NPC3. *:*p *<0.05; **:*p *<0.01; ****: *p* <0.0001.

Hydrogen peroxide (H_2_O_2_) plays a critical role in signal transduction and is regulated enzymatically [28, 29, 30]. Due to the increased H_2_O_2_ production in TBI [31], we examined expression changes in catalase (CAT), glutathione peroxidase 1 (GPx1), and the neuronal and astrocytic isoforms of peroxiredoxin (Prx5 and Prx6, respectively) [32, 33] via immunoblotting in the ipsilateral regions post‐CCI. We hypothesized that oxidative burden would increase enzyme translation and NPC3 treatment would restore expression levels to baseline. We observed an increase in CAT and Prx6 in the ipsilateral cortex (CX) and hippocampus (HC), respectively, which significantly differed from CCI+NPC3 (Figure 3A–C). Additionally, heme oxygenase 1 (HO1), a downstream marker for the activation of the nuclear factor erythroid 2‐related factor 2 (Nrf2) pathway [34, 35], peaked at 1 day post‐CCI, which was restored following NPC3 treatment. Expression levels for CAT, Prx6, and HO1 positively correlated with GFAP expression at 1 day post‐CCI in the ipsilateral cortex (Figure 3D). No significant differences between CCI and CCI+NPC3 were observed for GPx1. However, Prx5 was reduced at each time point for both treatment groups and negatively correlated with GFAP expression. Overall, these results suggest the antioxidant capacity of NPC3 may influence changes in the expression and activity of antioxidant defense systems post‐CCI.

### 3.4 NPC3 Intervention Acutely Influences Metabolic Function Post‐CCI

**FIGURE 4:**
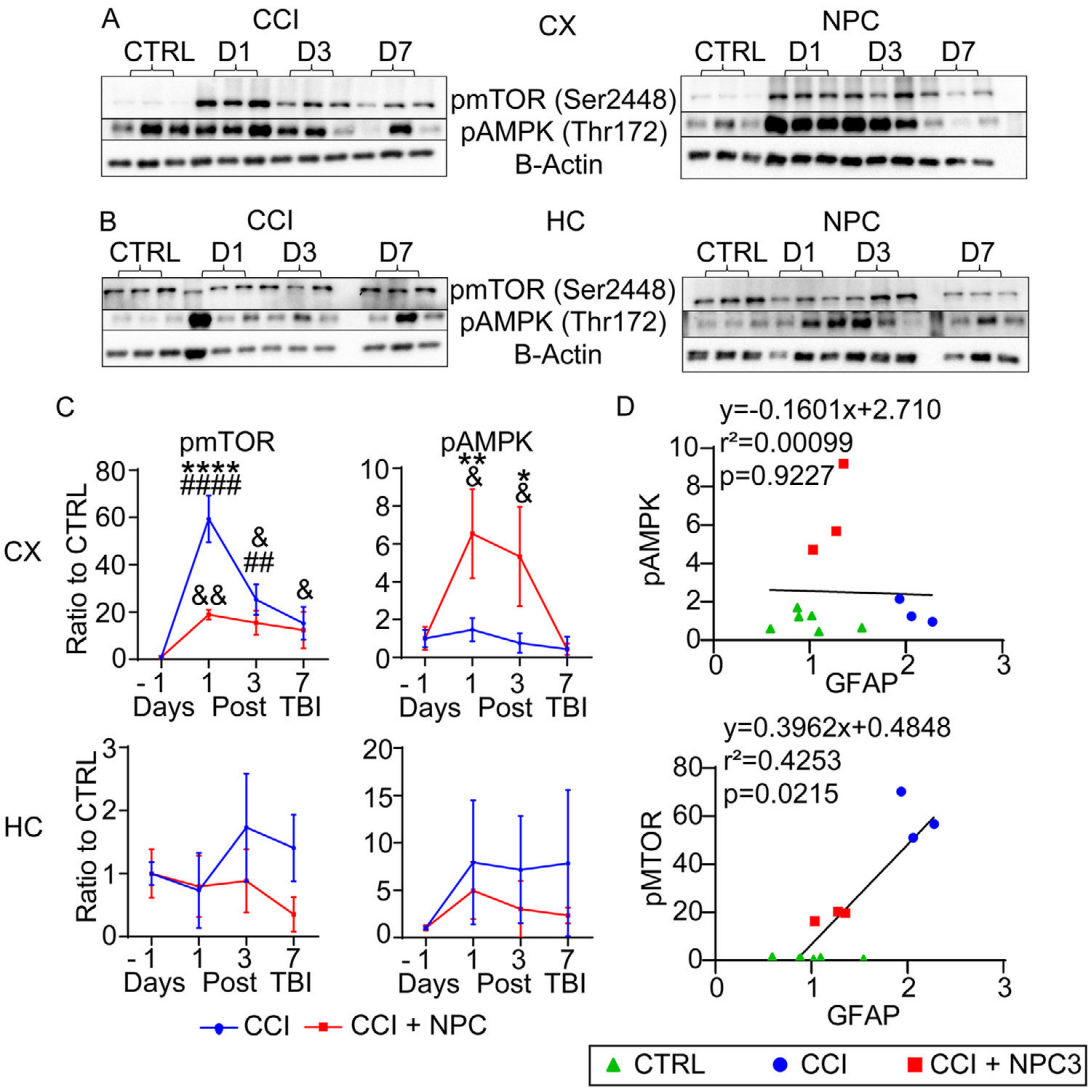
Effect of NPC3 intervention on cellular metabolism post 2.5 mm depth CCI. Western blot images of markers of cellular metabolism from the (A) ipsilateral cortex (CX) and (B) hippocampus (HC). (C) mTOR phosphorylation (Ser2448) peaked at 1 day post‐CCI and significantly differed from CCI+NPC3. AMPK phosphorylation (Thr172) significantly increased from CCI at 1 and 3 days following NPC treatment. (D) mTOR phosphorylation positively correlated with GFAP expression. No correlation between AMPK phosphorylation and GFAP expression was observed. Data are represented as mean ± SD (N = 3/treatment/day; CTRL samples were used for both CCI and CCI+NPC3 blots; N = 21 total). #: CTRL vs CCI; &: CTRL vs CCI + NPC3; *:CCI vs CCI + NPC3. *:*p* <0.05; **:*p *<0.01; ****: *p* <0.0001.

Injury sequalae generate an energy imbalance in TBI, contributing to glucose metabolism disruption, mitochondrial dysfunction, and an overall disruption in ATP production [36, 37, 38, 39]. Given the association between redox homeostasis and protein kinase activity [40, 41], we examined changes in the phosphorylation of the mechanistic target of rapamycin complex 1 (mTORC1) and adenosine monophosphate‐activated protein kinase (AMPK). We observed a significant increase in mTORC1 phosphorylation (Ser2448) [42] at 1‐day post‐CCI in the ipsilateral cortex, which was significantly reduced with NPC3 treatment (Figure 4A–C). Additionally, NPC3 treatment was associated with an increase in AMPK phosphorylation (Thr172) at 1 and 3 days post‐CCI. Phosphorylation of mTORC1 in the ipsilateral cortex positively correlated with GFAP expression at 1 day post‐CCI, while no significant correlation was observed between GFAP and AMPK phosphorylation (Figure 4D).

### 3.5 NPC3 Moderates CCI Generated Structural Brain Damage as Observed Using MRI

**FIGURE 5:**
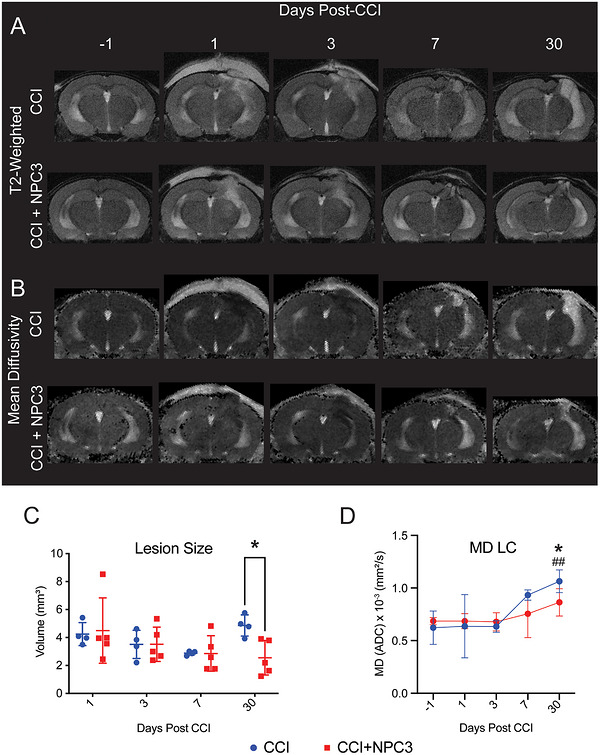
Effect of NPC3 intervention on lesion size and diffusion MRI metrics post 2.5 mm depth CCI. (A) Representative T2‐weighted images of the same mouse pre‐CCI and four timepoints post‐CCI. (B) At 30 day post‐CCI, the lesion volume was significantly smaller with NPC3 treatment. (C) Representative images of mean diffusivity of the same mouse pre‐CCI and four time points post‐CCI. (D) At 30 day post‐CCI, the mean diffusivity of the left cortex was significantly reduced with NPC3 treatment. Data are represented as mean ± SD (N = 4, CCI; N = 5, CCI + NPC3, longitudinal study). #: CCI Day −1 vs CCI; and: CCI+NPC3 Day −1 vs CCI + NPC3; *:CCI vs CCI+ NPC3. *:*p *<0.05.

The antioxidant activity of NPC3 acutely reduced astrocyte reactivity and affected both antioxidant defense and metabolism, suggesting a therapeutic effect post‐CCI. Thus, we investigated the efficacy of NPC3 in mediating structural changes prior to and following impact (baseline (‐1 day), 1 day, 3 days, 7 days, and 30 days post‐CCI). T2‐weighted MRI revealed an increase in voxel intensity at the injury site at 1 day post‐CCI (Figure 5A). By day 7, the intense region gave way to a cavity, which by day 30 had filled with fluid, as observed by the hyperintense lesioned region (Figure 5C). Indeed, the presence and development of an edemic fluid‐filled cavity was evident in the mean diffusivity (MD) measurements post‐CCI (Figure 5D). However, NPC3‐treated mice showed a significant reduction in lesion size and a significantly lower MD at day 30, as compared to untreated mice (Figure 5D). The complete image analysis of the T2‐weghted MRI and MRI metrics is available in Figures S5–S13.

### 3.6 Tractography of Ex Vivo Samples at 6 Months Post‐CCI Reveals Tract Variation but Minimal Difference From NPC3 Intervention

**FIGURE 6:**
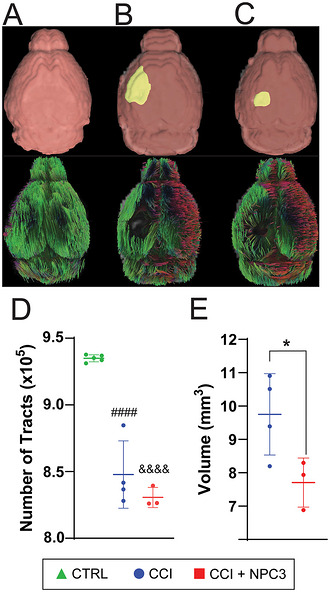
Effect of NCP3 intervention on diffusion tractography and lesion size post 2.5 mm depth CCI. Representative whole brain seeding region of interest for tractography images from (A) CTRL, (B) CCI, and (C) CCI+NPC3. (D) The number of tracts reveals a significant difference *p* <0.0001 between CTRL with CCI and CCI+NPC3. As shown in (E), a significant difference was observed in the lesion volume between CCI and CCI+NPC3. Data are represented as mean ± SD (N = 5, CTRL, N = 4, CCI; N = 3, CCI + NPC3) #: CCI vs. CTRL, & CTRL vs CCI+NPC3, CCI vs CCI + NPC3; *:*p *<0.05; ****:*p *<0.0001.

Due to the neuroprotective effect of NPC3 on mediating structural defects post‐CCI, we evaluated changes in axonal tracts using diffusion tractography at 6 months post‐CCI. Diffusion tractography measures the movement of water between locations and generates a map, which can be used to estimate the microstructural integrity of white and grey matter in the brain [43]. After applying the same seeding density to all the ex vivo brains, representative tractography images and corresponding region of interest maps revealed a noticeably smaller lesion volume with NPC3 treatment (Figure 6A–C). Indeed, while the number of tracts did not differ between CCI and CCI+NPC3, we did observe a significant reduction in lesion volume with NPC3 treatment (Figure 6D,E), corresponding with T2‐weighted MRI results.

### 3.7 NPC3 Alleviates Glial Activity and Urinary 8‐Isoprostane at Moderate Impact Severity

**FIGURE 7:**
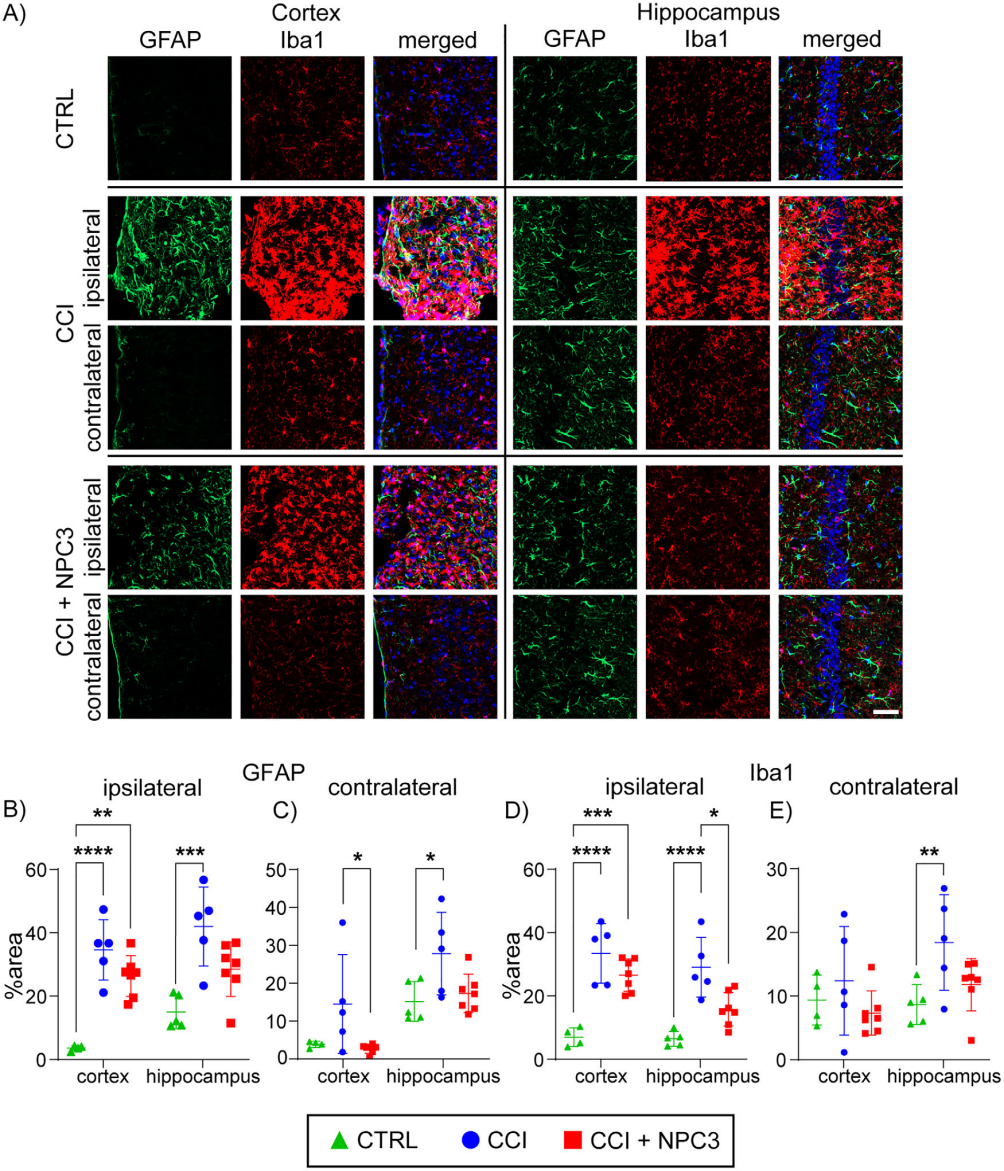
Quantification of GFAP and Iba1 in cortex and hippocampus at 7 days post 1.5 mm depth CCI. (A) Representative confocal images of control, CCI, and CCI+NPC3 treatment groups in the bilateral cortex and hippocampus. Scale bar corresponds to 50 µm. Quantification of GFAP (B, C) and Iba1 (D, E) cell area in the bilateral cortex and hippocampus. NPC3 treatment reduced the reactivity of astrocytes and microglia bilaterally at 7 days post‐CCI. Data are shown as mean ± SD. Each point represents an individual mouse with N = 4, control cortex; N = 5, control hippocampus; N = 5, CCI; N = 7, NPC. *, **, ***, and **** indicate a statistical difference of *p* < 0.05, *p* < 0.01, *p* < 0.001, and *p* < 0.0001, respectively, as determined by two‐way ANOVA and Tukey's post hoc test.

**FIGURE 8:**
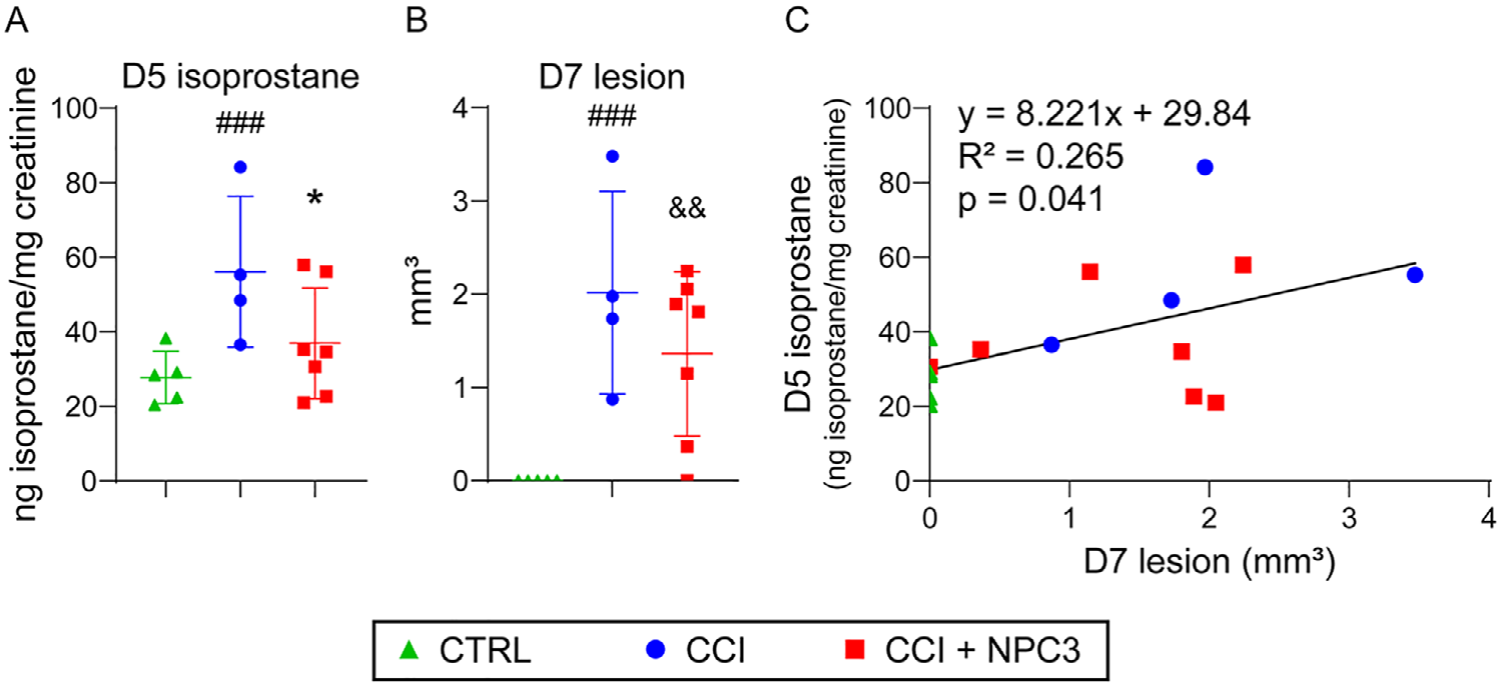
Correlation between urinary 8‐isoprostane concentration at 5 days and lesion size at 7 days post 1.5 mm depth CCI. (A) Urinary 8‐isoprostane concentration at 5 days post‐CCI as measured by ELISA. (B) Lesion size at 7 days post‐CCI as measured by T2‐images. Data are shown as mean ± SD (N = 5, control; N = 4, CCI; N = 7, NPC). #: CTRL vs CCI; &: CTRL vs CCI+NPC3; *: CCI vs CCI+NPC3 *:*p *<0.05; **:*p* <0.01; ***: *p* <0.001, as determined by one‐way ANOVA and Tukey's post hoc test. (C) Correlation plot between urinal 8‐isoprostane concentration at 5 days and lesion size at 7 days post‐CCI. There is a strong correlation (*p* < 0.05) between the reduction in the 8‐isoprostane concentration on day 5 and the lesion size on day 7 with NPC3 treatment, as determined by simple linear regression.

Our previous results demonstrated that NPC3 alleviated astrocyte activation, influenced redox metabolism, and mediated the progression of a lesion to a fluid‐filled cavity in a severe CCI model (2.5 mm depth). Thus, we extended our study to examine the therapeutic efficacy of NPC3 in a moderate severity of CCI (1.5 mm depth). Reducing the severity of CCI aids in assessing a therapeutic effect by reducing the level of necrotic damage to the hippocampus resulting from the primary impact, rather than secondary spread of damage [24]. T2‐weighted images revealed no significant differences in lesion or cavity size between CCI at 1.5 mm depth and the CCI + NPC3 at day 1(Figure SF1), corresponding with our findings using the 2.5 mm depth. However, we observed a trending reduction in GFAP and Iba1, two markers of neuroinflammation, with NPC3 treatment in the bilateral cortex and hippocampus (CA1) at 7 days post‐CCI (Figure 7A–E, Figure SF2). In the contralateral hemisphere, although we did not observe significant differences, NPC3 treatment did mitigate the overexpression of GFAP and Iba1 compared to CTRL (*p* ≥ 0.59) (Figure 7C,E). NPC3 treatment at 5 days post‐moderate CCI reduced levels of LPOx, as measured by a reduction in urinary 8‐isoprostane concentration [44] (Figure 8A, Figure SF3). Additionally, treatment with NPC3 resulted in a trending reduction in lesion size at 7 days post‐CCI (Figure 8B). Indeed, we found a strong correlation between the reduction in 8‐isoprostane concentration at D5 and lesion size at D7 (p < 0.05) with NPC3 treatment (Figure 8C), further supporting our MRI findings of delayed progression of injury.

## 4 Discussion

Oxidative and electrophilic stress exacerbate TBI pathophysiology, resulting in neuropathological features and behavioral deficits. NPC3 is an antioxidant NP treatment designed to scavenge ROS and LPOx, two stressors that play a prominent role in TBI sequalae. By scavenging these components, NPC3 joins the long list of antioxidant small‐molecule drugs and NP designed for use against the secondary effects of TBI [18, 45]. Our previous results demonstrated IV administration of NPC3 accumulated within the perilesional region acutely post‐CCI and improved behavioral outcomes [21, 22]. Indeed, CCI mice treated with NPC3 showed improved spatial learning and memory [22] as measured by increases in escape frequency and reduced primary latency in the Barnes Maze [22, 46]. While these improved outcomes suggest an amelioration in neuropathological features post‐CCI, we have yet to monitor lesion progression; thus, limiting our understanding of antioxidant NP function in TBI.

MRI showed structural changes in the brain following CCI including the formation of an initial diffusion restricted lesion followed by progression into a fluid‐filled free diffusion cavity, a common observation of edema following impact [47, 48, 49, 50]. This fluid restriction is considered to be cytotoxic edema in the acute phase, which subsequently evolves into increased diffusion vasogenic edema in the sub‐acute phase and finally becomes the characteristic fluid‐filled cavity in the chronic phase of the injury [51]. The progressive lesion expansion is a hallmark of secondary injury and chronic microglial activation [52]. NPC3 treatment slowed the progression of the lesion from cytotoxic edema to vasogenic edema and reduced the size of vasogenic edema and the fluid‐filled cavity. Sharma et al. also observed a reduction in lesion volume via immunomodulatory PLGA‐COOH NP through MRI tracking, which they attributed to a reduction in infiltrating immune cells, astrocyte reactivity, and microglia activation [53, 54]. Antioxidant NP have also shown a reduction in astrocyte reactivity and microglia activation in the CCI mouse model of TBI [18]. This delay in lesion progression to a fluid‐filled cavity suggested the therapeutic potential of NPC3 in neuroprotection, potentially through mechanisms related to reducing cell death caused by cytotoxic edema and reducing neuroinflammation, both of which can lead to BBB protection and vasogenic edema reduction. However, because BBB integrity was not directly addressed, these findings should not be interpreted as direct evidence of BBB protection.

NPC3‐mediated reduction in vasogenic edema was associated with a reduction in neuroinflammation, as we observed decreases in both GFAP and Iba1 expression in CCI mice treated with NPC3 (Figures 2 and 7, Figure SF2). Astrocyte reactivity and proliferation increase in response to a variety of biochemical stimuli, including ROS, resulting in the upregulation of endogenous antioxidant defense systems to protect neurons, including increased glutathione synthesis and Nrf2 activation [55]. Due to the antioxidant capacity of NPC3 treatment, coupled with the reduction in GFAP observed previously, we hypothesized that NPC3 would reduce enzymatic translation and antioxidant system activity post‐CCI. Expression increases in CAT, Prx6 and HO1 post‐CCI positively correlated with GFAP in the ipsilateral cortex and were reduced with NPC3, suggesting NPC3's antioxidant capacity may influence redox‐regulated transcriptional/translational changes in antioxidant defense. Indeed, these results corroborate with previous literature demonstrating Nrf2 activation in TBI [56, 57, 58]. However, while previous therapeutic strategies have attempted to augment the Nrf2 pathway [59, 60], our results suggest thiol‐based antioxidant treatments may reduce Nrf2 activity through alleviating oxidant burden. These findings are further supported by urinary isoprostane results demonstrating LPOx levels were reduced to baseline following NPC3 treatment (Figure 8, Figure SF3). Interestingly, NPC3 treatment differentially regulated the expression of CAT in the cortex and HC, conflicting with our original hypothesis. These results may be explained by the delayed increase in HO1 expression observed at day 3 following NPC3 treatment, as Nrf2 activation plays a role in regulating CAT [61, 62]. Indeed, we observed a trending increase in each antioxidant marker at 3 days following NPC3 treatment in both the cortex and hippocampus (Figure 3). The delayed increase in antioxidant defense at day 3 may result from a reduction in necrotic damage via NPC3 [22, 24], providing a recovery in endogenous activity. However, future studies should examine transcription level changes in enzyme expression following NPC3 treatment. Additionally, we observed a significant decrease in Prx5 in the ipsilateral cortex, which has been observed previously in a model of subarachnoid hemorrhage [63]. Prx5 is expressed predominantly in neurons [33], and negatively correlated with GFAP expression, suggesting the antioxidant effect of NPC3 may preferentially protect astrocytes. However, future studies are needed to identify the cell‐specific effect of NPC3.

TBI sequelae generate an energy imbalance resulting from perturbed glucose metabolism, mitochondrial dysfunction, and impaired ATP production [37, 64, 65]. Moreover, alterations in redox homeostasis have been associated with changes in protein kinase signaling [40, 66, 67]. Thus, we examined changes in phosphorylation for mechanistic target of rapamycin complex 1 (mTORC1) and adenosine monophosphate‐activated protein kinase (AMPK). Due to the role of mTORC1 as a regulator of cellular metabolism, increased activation may occur in response to altered energy or amino acid availability. Increased mTORC1 phosphorylation (Ser2448) at 1 day post‐CCI in the ipsilateral cortex correlated with GFAP, supporting an association between mTOR signaling and astrocyte reactivity following injury [68, 69]. Indeed, reduced mTORC1 phosphorylation observed following NPC3 treatment is consistent with previous literature demonstrating altered mTOR signaling in TBI and therapeutic benefit following pharmacological inhibition [70, 71, 72]. Additionally, we observed an increase in AMPK phosphorylation (Thr172) at 1 and 3 days post CCI following NPC3 treatment. However, phosphorylation levels did not correlate with GFAP levels. Overall, these findings demonstrate that NPC3 treatment was associated with altered mTORC1 and AMPK phosphorylation post‐CCI. However, because our study did not examine upstream or downstream signaling pathways, future studies are needed to determine the mechanistic significance of these findings and elucidate factors influencing mTOR/AMPK activity following CCI.

White and grey matter integrity is often disrupted in TBI and diffusion MRI techniques can provide insight into disease progression. By assessing the movement of water molecules, there is evidence of structural and microstructural changes in TBI, especially in a mouse model [73]. Indeed, diffusion MRI studies have been conducted in CCI models of TBI, such as mice, rats, and ferrets [73, 74, 75, 76, 77]. These studies report varying amounts of disruption to the tracts of their respective animal model. Damage in the ex vivo samples presented in Figure 6 similarly shows a lack of tract formation in the lesion area. As shown in our tractography models, the difference between the CCI and CCI+NPC3 did not have significant differences in the number of tracts. However, NPC3 treatment did result in a decrease in lesion volume (*p* <0.05). Here, a whole brain seeding technique provided evidence of tract loss in the injured region, which in addition to traditional DTI analysis can be correlated with lower fractional anisotropy (FA) [78]. A decrease in FA is nonspecifically and often associated with a loss in axonal integrity. In both clinical and preclinical studies, changes in FA can indicate potential changes in the myelination of white matter or possible developments of edema [79, 80]. Even in a CCI study that evaluated up to one year post‐impact, a reduction in FA was observed, similar to our 6 month post‐injury evaluation [47].

Overall, these results suggest NPC3 provides a therapeutic effect in severe CCI. However, due to the impact heterogeneity associated with clinical TBI, there is a critical need to evaluate therapeutic strategies in multiple injury severities to verify their efficacy. Indeed, severe CCI presents with increased levels of necrotic damage to the hippocampus, which are not observed in the majority of cases clinically [24, 81]. Thus, we examined the effect of NPC3 in alleviating secondary damage associated with moderate CCI (1.5 mm depth). We found a trending reduction of GFAP and Iba1 with NPC3 treatment in the bilateral cortex and hippocampus (CA1) at 7 days post‐CCI (Figure 7A–E, Figure SF3). Additionally, NPC3 treatment reduced urine levels of 8‐isoprostane at 5 days post‐moderate CCI (Figure 8A, Figure SF4). Increased levels of 8‐isoprostane are clinically associated with more severe morbidity and mortality outcomes [82], suggesting a reduction in 8‐isoprostane via antioxidant treatment may infer a therapeutic effect. These findings strongly correlated with the NPC3‐mediated reduction in lesion size at 7 days post‐CCI (Figure 8B,C), further supporting our Western blot and MRI findings of delayed progression of injury. Thus, these results support the role of NPC3 in alleviating the injury sequalae presented along the continuum of TBI severities. However, due to the lack of significant reduction in acute lesion volume (i.e., days 1–7), future studies should investigate NPC3's efficacy in alleviating molecular changes at sub‐acute and chronic timepoints to correlate with the observed moderation of structural deficits.

Within the scope of this study, there are several limitations that should be noted. As demonstrated in our prior work, the selected dose (8 mg/kg) and immediate post‐impact administration were determined based on our previous study evaluating treatment window and dose‐response relationships [22]. However, assessing the delivery of a single dose without delayed administration limits translational insights regarding therapeutic efficacy as oftentimes TBI patients do not begin treatment until hours after initial injury. Another limitation is that only male mice were used in this follow‐up study, which was by design since we previously identified a sex‐based dependency on the dose and efficacy of NPC3 treatment in CCI [22], where treatment was shown to have a greater therapeutic potential in male mice. Additionally, the use of naïve mice may not be adequate to serve as a control for CCI mice, as craniectomy alone has been observed to cause structural, molecular, and behavioral deficits [83, 84, 85]. Indeed, because a primary objective of this study was to evaluate NPC3 treatment efficacy using MRI‐based structural outcomes, naïve mice were used as controls, as our previous work demonstrated that craniectomy resulted in a protrusion of the brain through the cranial window that may complicate our interpretation of lesion and cavity formation [85]. An additional limitation of this study is the relatively small sample size for animal experiments, which may not be sufficiently powered to detect important differences between CCI and NPC3 treated mice. However, power analysis revealed that the number of mice was adequate for observing statistical differences between CTRL and CCI samples, providing relevant signal for observing trends in therapeutic efficacy. Indeed, even with small sample sizes (i.e., N = 3), we were able to observe significant differences between the naïve, CCI, and CCI+NPC3 treatment groups. Additionally, using non‐invasive longitudinal MRI studies and time‐course changes in protein expression, our data highlight the potential efficacy of NPC3 as a treatment strategy in TBI and highlight the need for future studies examining their mechanism of action. Finally, we did not directly test BBB integrity but rather relied on radiographic findings and their typical clinical interpretation for this observation. Nor did we consider the possibility of peripheral immune cell activation and infiltration into the injury as our study focused on changes in neural cells.

In summary, our results suggested that NPC3 reduces the progression of TBI‐induced cerebral edema by alleviating ROS‐mediated vasogenic edema caused by neuroinflammation and altered cell energy balance. The function of NPC3 was related to the antioxidant effects, which acts at least partly through restoring antioxidant defense and the Nrf2 signaling pathway, showing the thiol‐based antioxidant treatment acutely restores redox metabolism and alleviates sub‐acute neuropathological features. Combined with our previous results, NPC3 is a potential candidate for TBI treatment and may play a role in addressing the secondary effects of TBI.

## Author Contributions

All authors were responsible for conceptualization and methodology. E.T, B.M, A.T., and A.P. were responsible for investigation, visualization, and formal analysis. A.C. and F.K were responsible for supervision and funding acquisition. E.C and B.M were responsible for writing the original manuscript, and all authors participated in reviewing and editing the final draft.

## Funding

This research was funded by the National Institute of Neurological Disorders and Stroke of the National Institutes of Health (R01NS109488) and the National Institute of General Medical Sciences of the NIH (T32 GM136593).

## Ethics Statement

All animal work reported here was approved by the Institutional Animal Care and Use Committee at the University of Nebraska – Lincoln under protocol number 2300.

## Conflicts of Interest

A.C. and F.K. are named inventors on patents protecting this antioxidant nanoparticle technology and use, and with A.T. are co‐founders of NanoPhylax seeking to translate this technology. The remaining authors declare that they have no known competing financial interests or personal relationships that could have appeared to influence the work reported in this paper.

## Supporting information




**Supporting File**: mabi70214‐sup‐0001‐SuppMat.pdf.

## Data Availability

The data that support the findings of this study are available from the corresponding author upon reasonable request.
