# Supplementary material for: Thiol‐Based Neuroprotective Copolymers Acutely Restore Redox Metabolism and Mediate Vasogenic Edema in a Mouse Model of Traumatic Brain Injury
Source: Macromol Biosci. 2026 Jul 21;26(7):e00642. doi: 10.1002/mabi.202500642 (PMC13387508; doi:10.1002/mabi.202500642)
Supplement: Supplementary file 1 — Supporting File: mabi70214‐sup‐0001‐SuppMat.pdf. [file MABI-26-e00642-s001.docx]

**Supplementary Information**

**Thiol-based neuroprotective copolymers acutely restore redox metabolism and mediate vasogenic edema in a mouse model of traumatic brain injury**

**Authors: Evan T. Curtis^1^, Brandon Z. McDonald^1^, Aria W. Tarudji^1^, Aaron M. Priester^2^, Anthony J. Convertine^2^, Forrest M. Kievit^1^***

**Affiliations:**

^1^ Department of Biological Systems Engineering, University of Nebraska-Lincoln, NE, 68583, USA

^2^ Department of Material Science and Engineering, Missouri University of Science and Technology, Rolla, MO, 65409, USA

**Corresponding Author:**

* Dr. Forrest M. Kievit

268 Morrison Center

4240 Fair Street, Lincoln, NE 68583-0900

fkievit2@unl.edu

**Supplementary Figures**


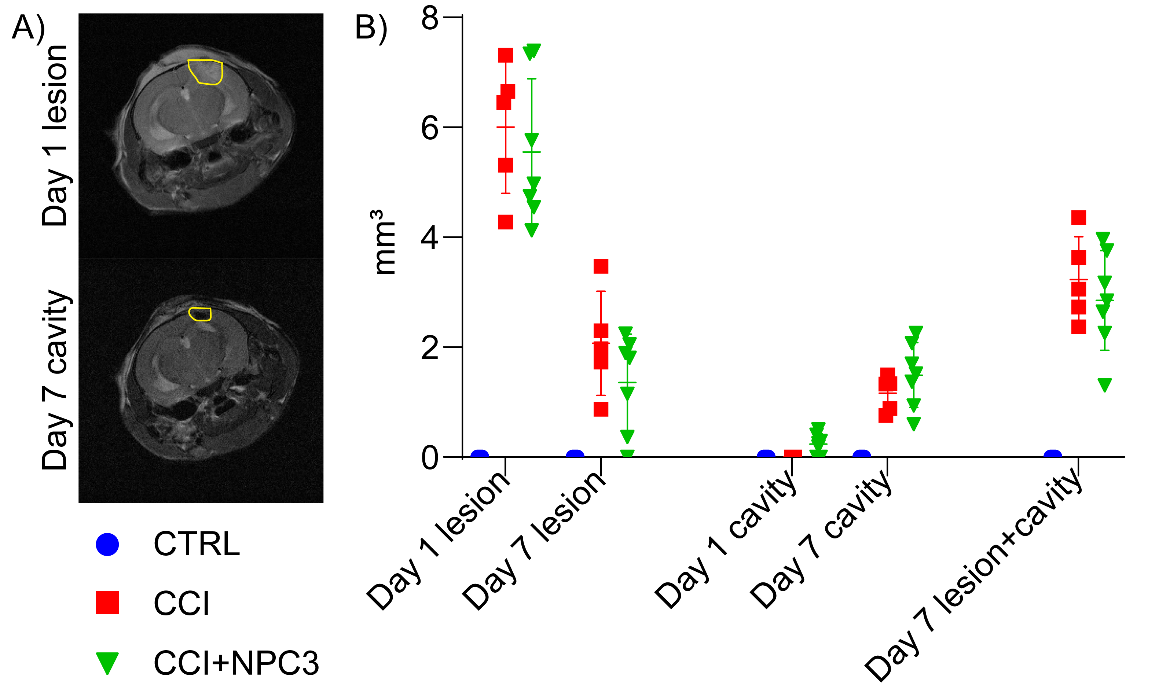


**Supplemental Figure 1.** Lesion and cavity volume taken with T2 imaging at 1.5mm impact depth CCI. A) Representative T2 mages of the ROI of the lesion and cavity on day 1 and 7 post-CCI, respectively. B) Lesion and cavity volume on day 1 and 7 post-CCI. NP treatment showed slight reduction in lesion on day 1 and 7 post-CCI. Data are shown as mean ± SD.


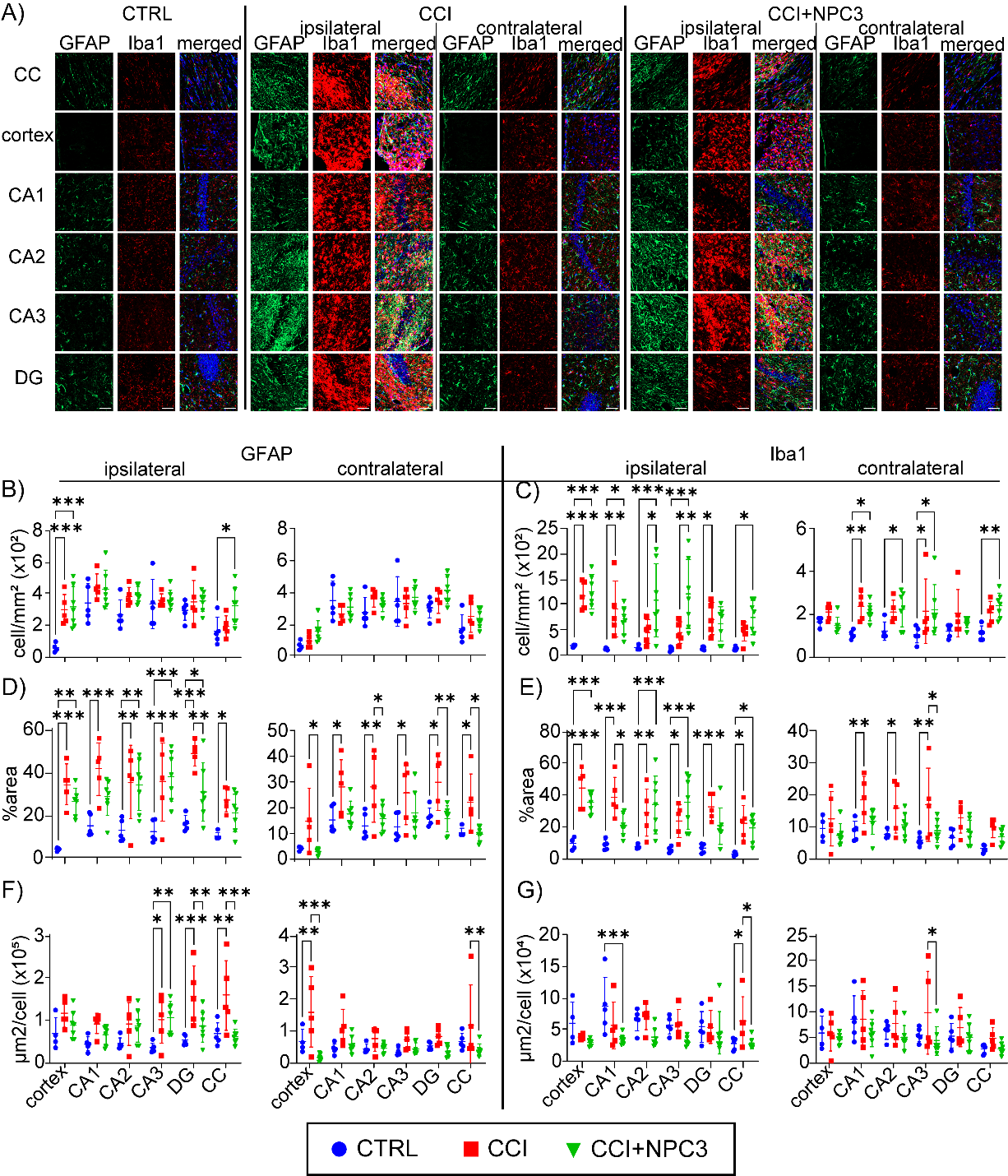


**Supplemental Figure 2**. Quantification of GFAP and Iba1 in the cortex, corpus callosum (CC), and hippocampus at 7 days post-CCI. A) Representative confocal image of bilateral cortex, CC, CA1, CA2, CA3, and dentate gyrus (DG) of control, CCI, and NP treated treatment groups. Scale bar corresponds to 50 µm. Quantification of GFAP (B,D,F) and Iba1 (C,E,G) positive cells (B,C), positive cell area (D,E), and average area per cell (F,G) in the bilateral cortex, CA1, CA2, CA3, DG, and CC. NP treatment reduced the spread of neuroinflammation into the contralateral hemisphere at 7 days post-CCI. Data are shown as mean ± SD. *, **, and *** indicate a statistical difference of p < 0.05, p < 0.01, and p < 0.001, respectively, as determined by two-way ANOVA and Tukey’s post hoc test. Data are shown as mean ± SD.


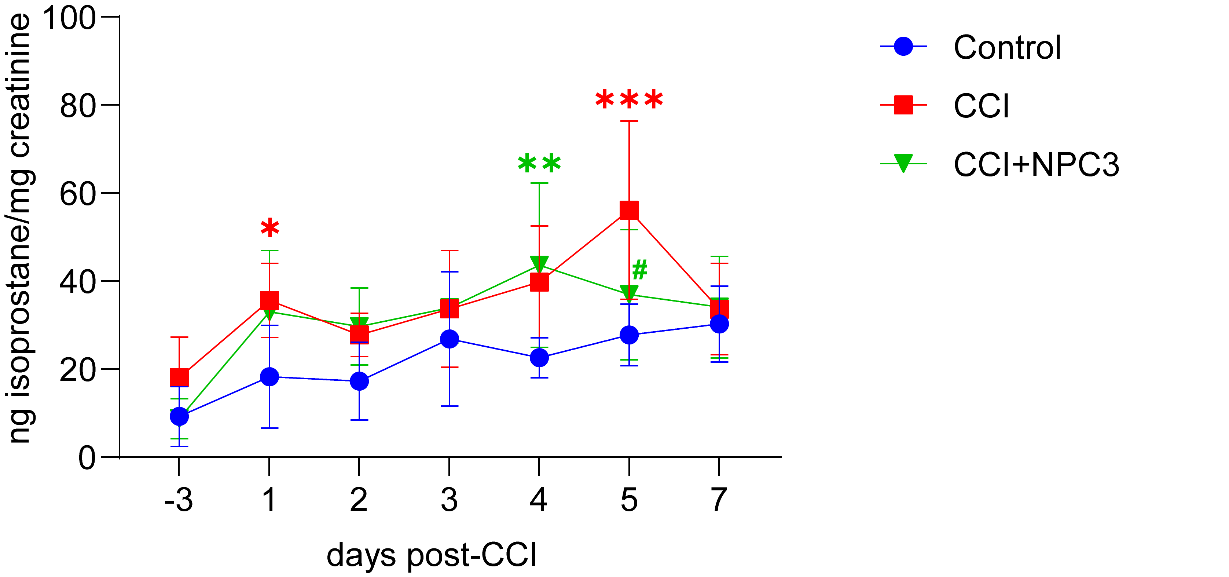


**Supplemental Figure 3**. Time-course of free 8-isoprostane concentration in the urine post-CCI. The concentration of 8-isoprostane was normalized to the concentration of creatinine in the urine. NP treatment did not reduce the elevation of isoprostane from CCI at day 1-4 post-CCI, but significantly reduce the isoprostane at day 5 post-CCI. * and # indicate a statistical difference as compared to control and untreated CCI, respectively, with one, two, and three symbols indicating p < 0.05, p < 0.01, and p < 0.001, respectively, as determined by two-way ANOVA and Tukey’s post hoc test. Data are shown as mean ± SD.

The figures presented below are a summary of the magnetic resonance imaging (MRI) data collected for *in vivo* analysis. All mice were evaluated at days -1 (baseline), 1 (Day1), 3 (Day 3), 7 (Week1), and 30 (Month1) post impact. The study numbers correspond to studies conducted in which T2-weighted images and diffusion tensor imaging (DTI) data were collected. DTI metrics, mean diffusivity (MD), fractional anisotropy (FA), and directional encoded color map (Color) were calculated and generated using DSI Studio. The summary includes study session and treatment condition (CCI or CCI+NPC3).


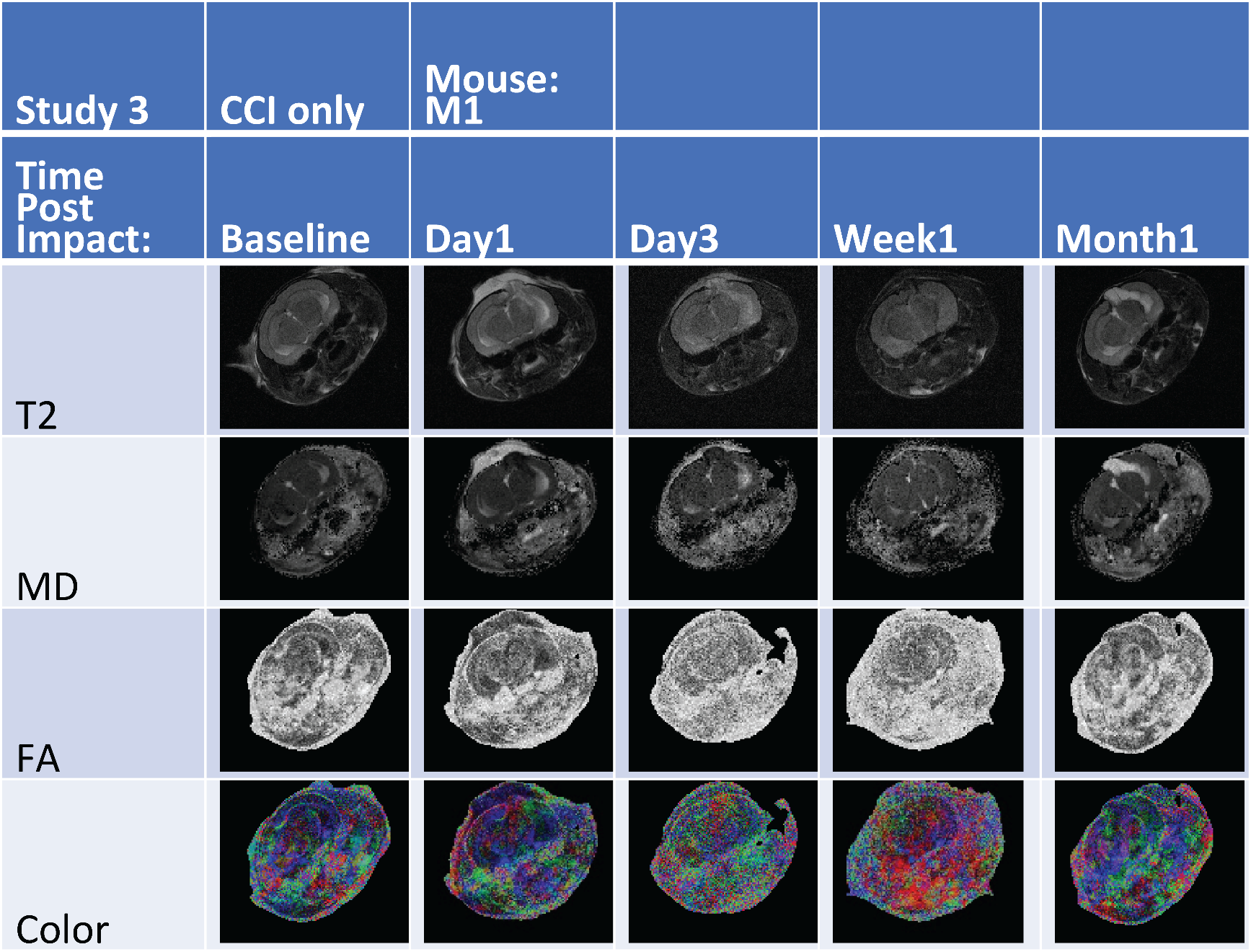


**Supplemental Figure 5**. Image summary of MRI sessions for Study3_Mouse1.


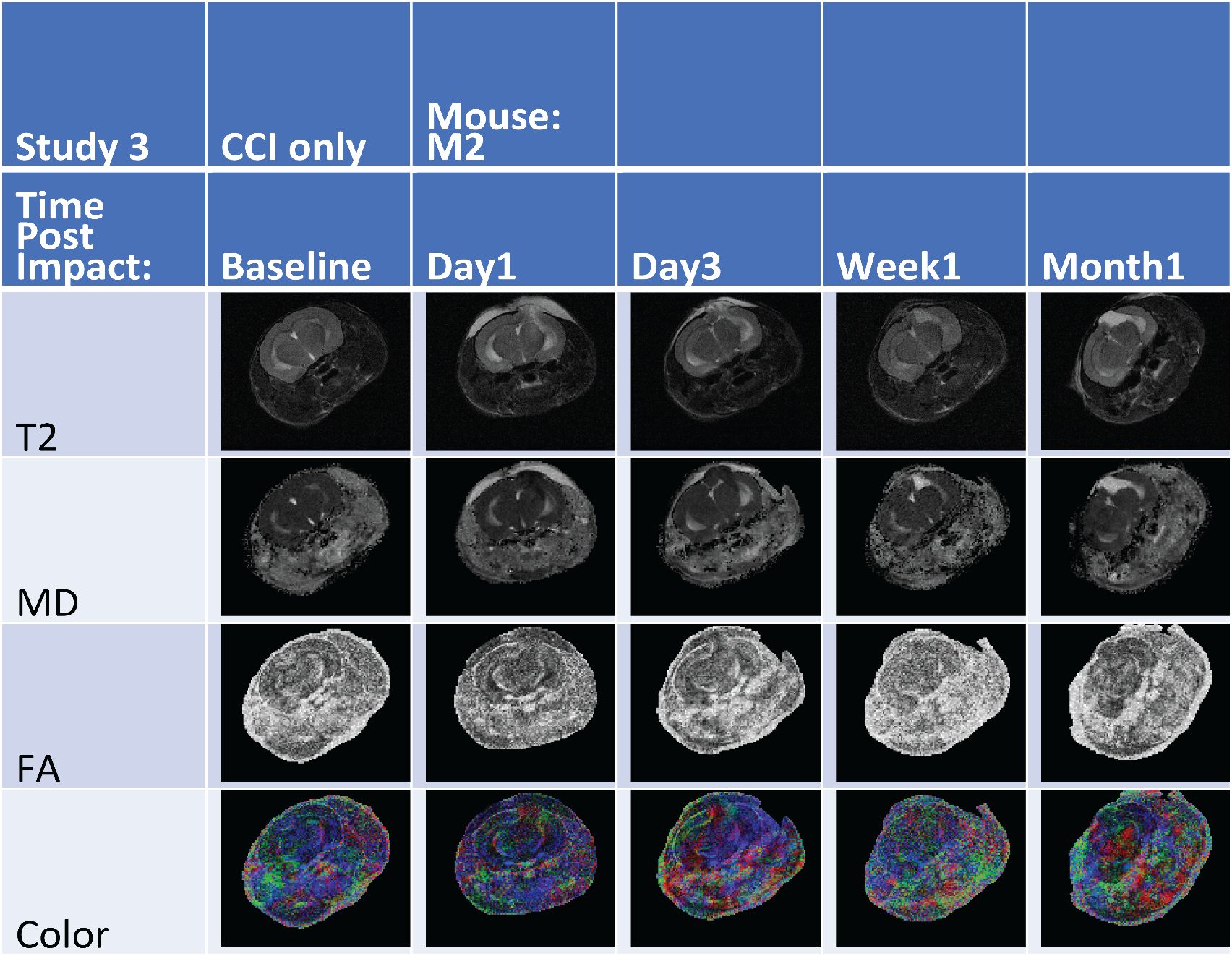


**Supplemental Figure 6**. Image summary of MRI sessions for Study3_Mouse2.


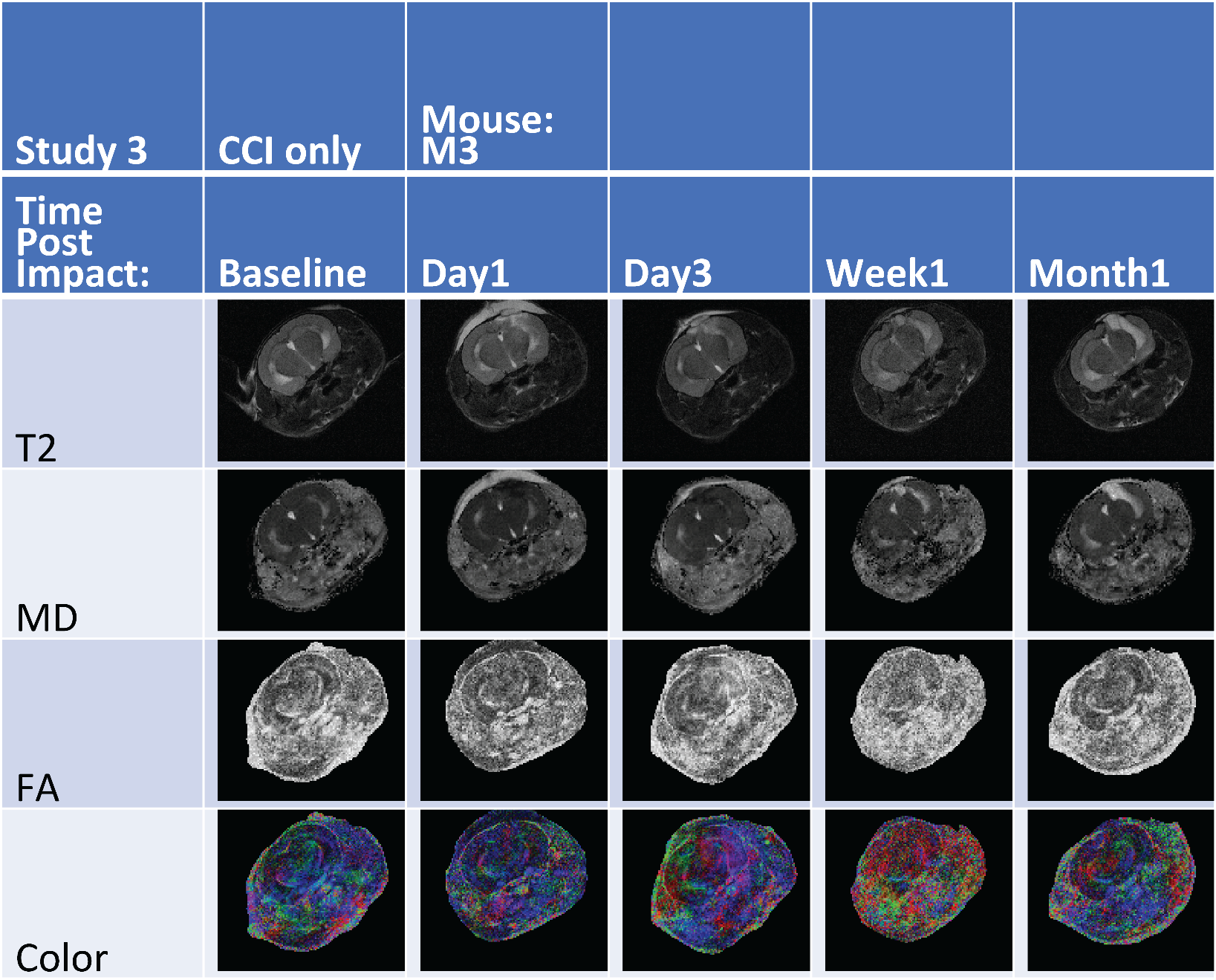


**Supplemental Figure 7**. Image summary of MRI sessions for Study3_Mouse3.


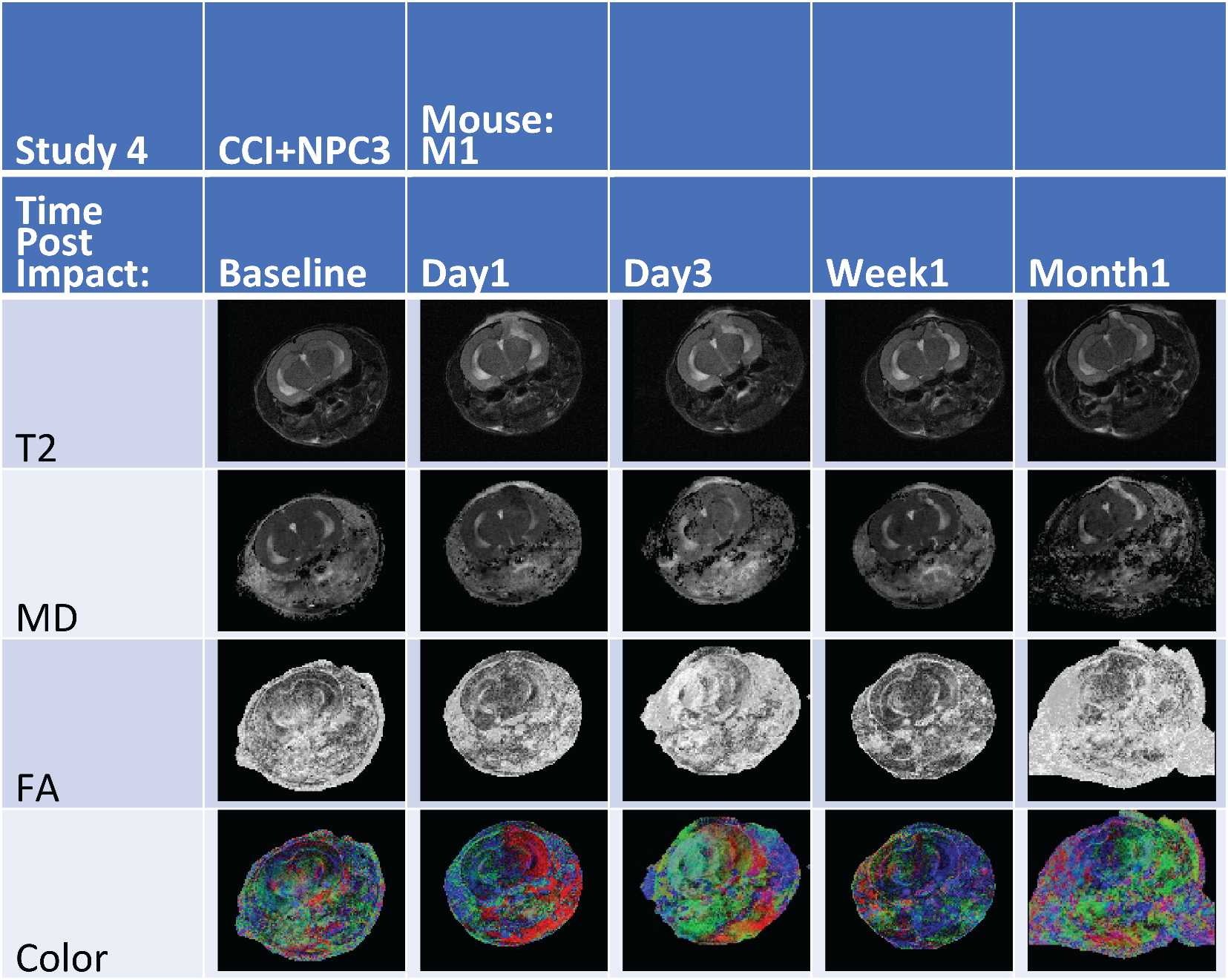


**Supplemental Figure 8**. Image summary of MRI sessions for Study4_Mouse1.


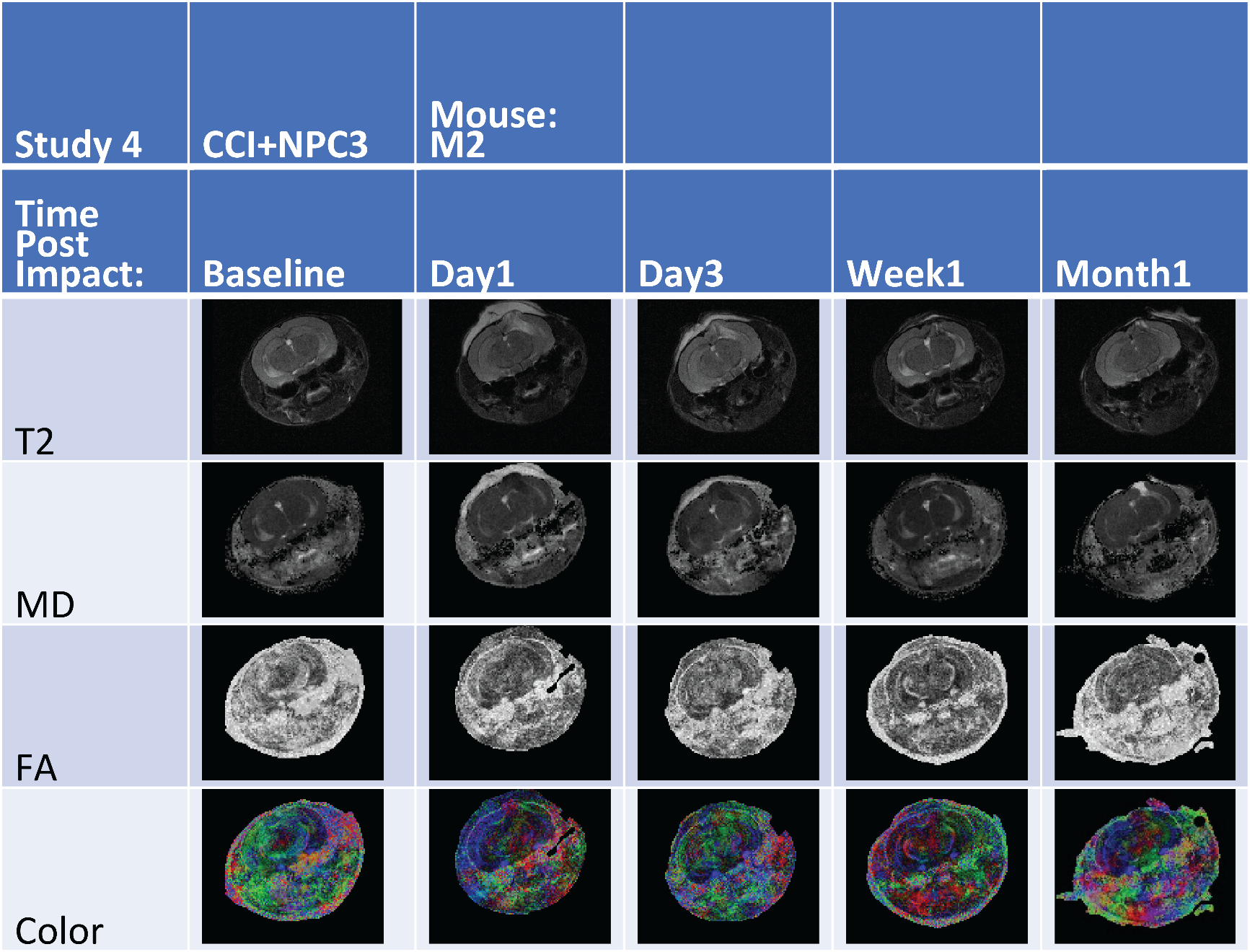


**Supplemental Figure 9**. Image summary of MRI sessions for Study4_Mouse2.


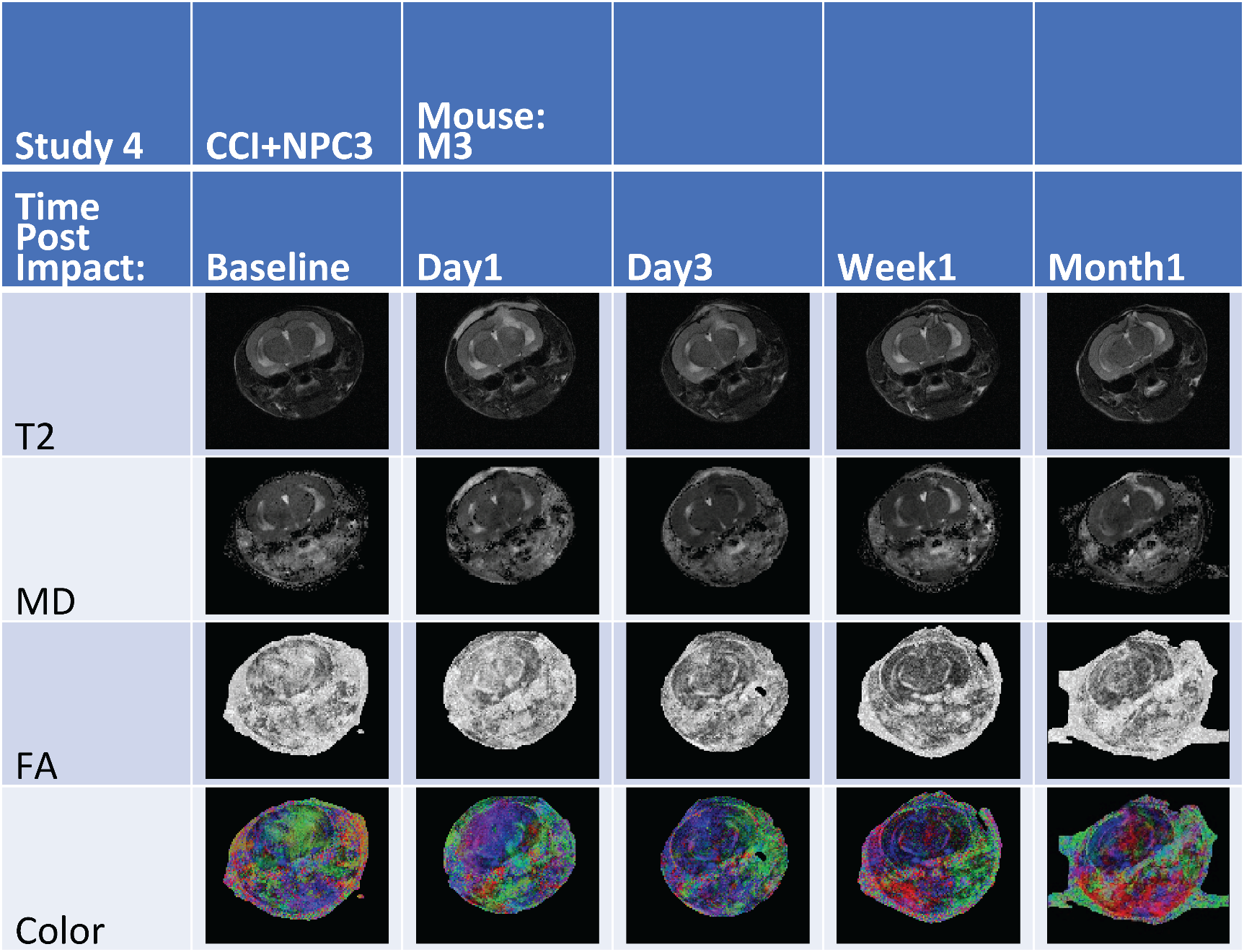


**Supplemental Figure 10**. Image summary of MRI sessions for Study4_Mouse3.


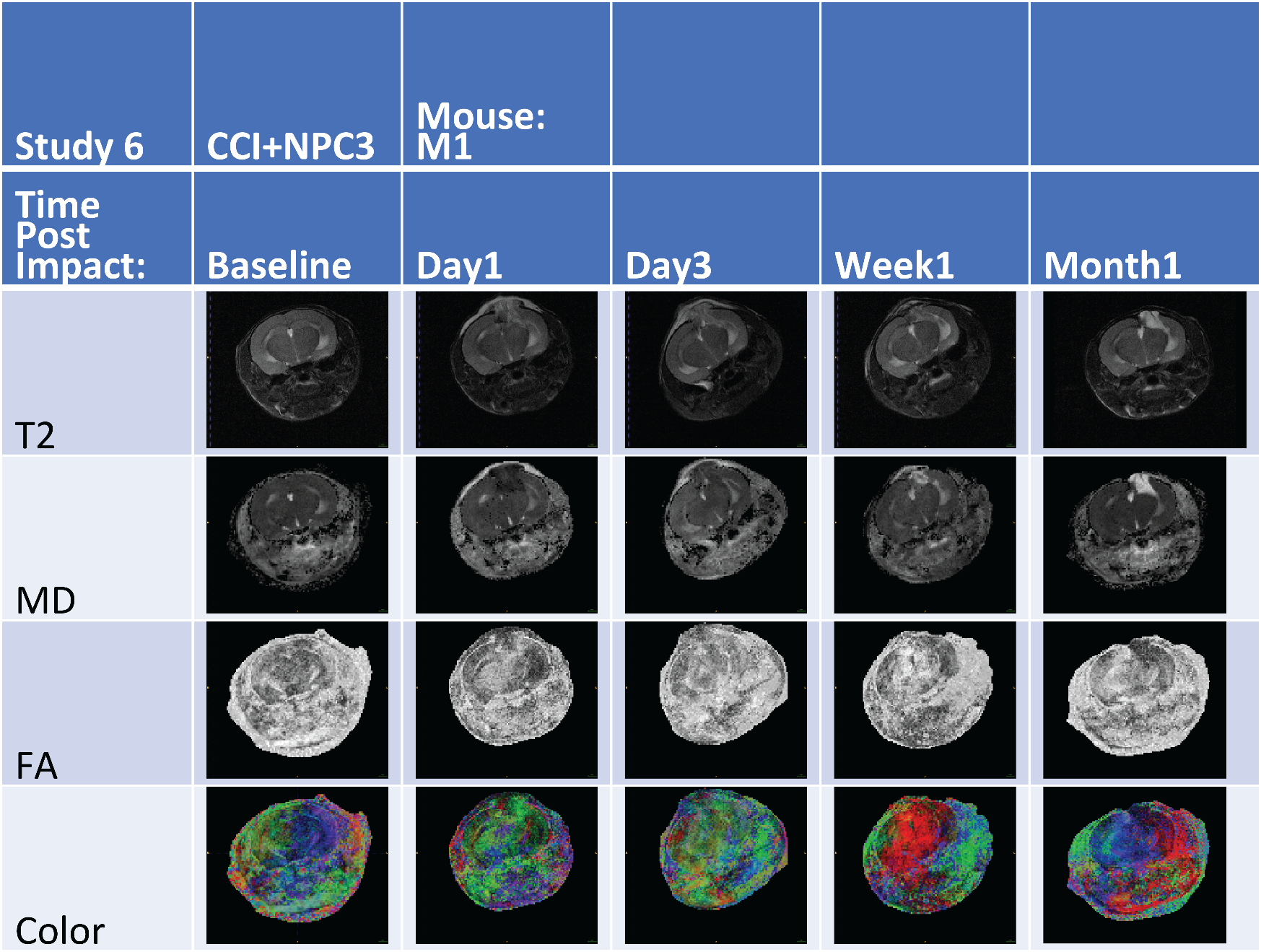


**Supplemental Figure 11**. Image summary of MRI sessions for Study6_Mouse1.


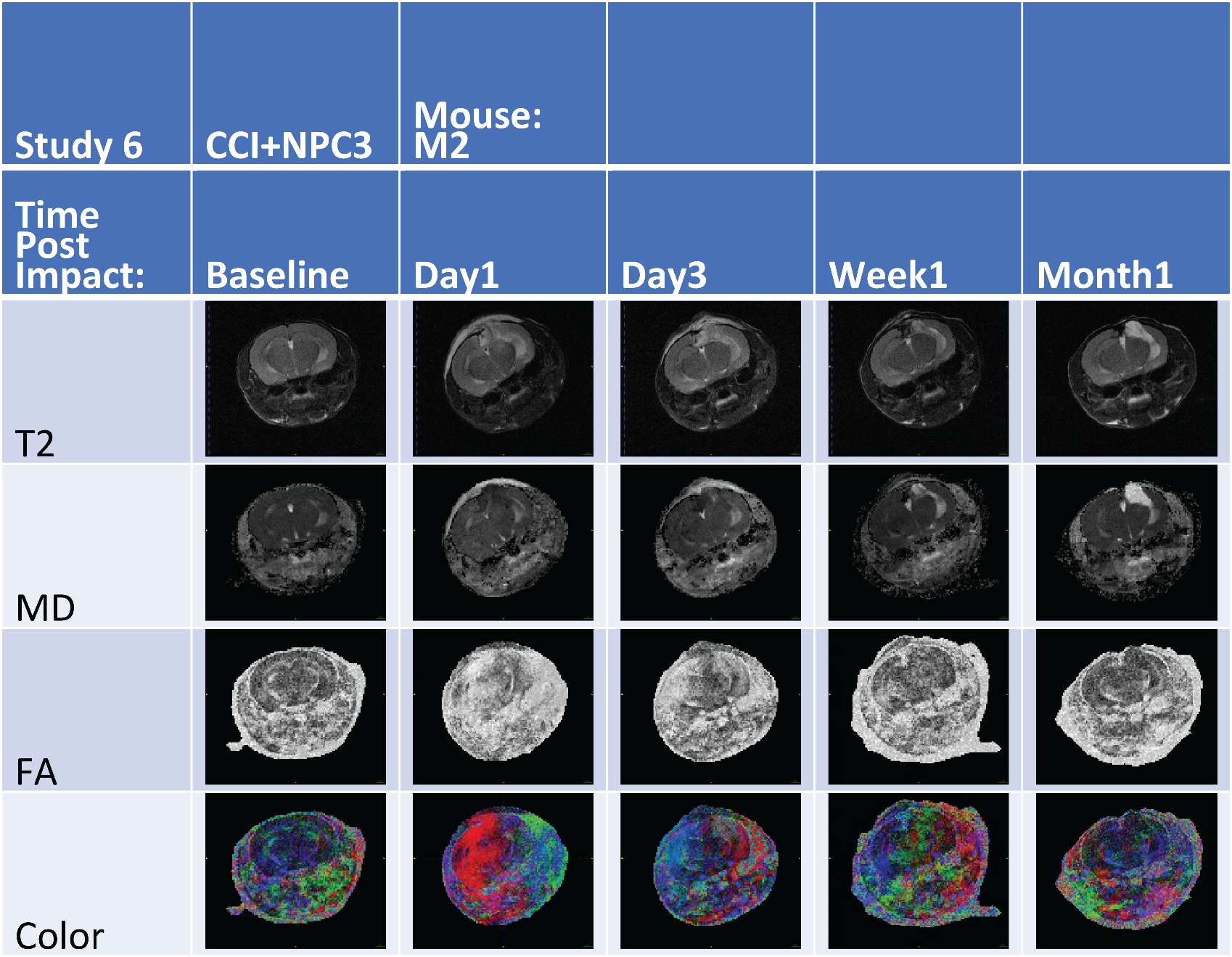


**Supplemental Figure 12**. Image summary of MRI sessions for Study6_Mouse2.


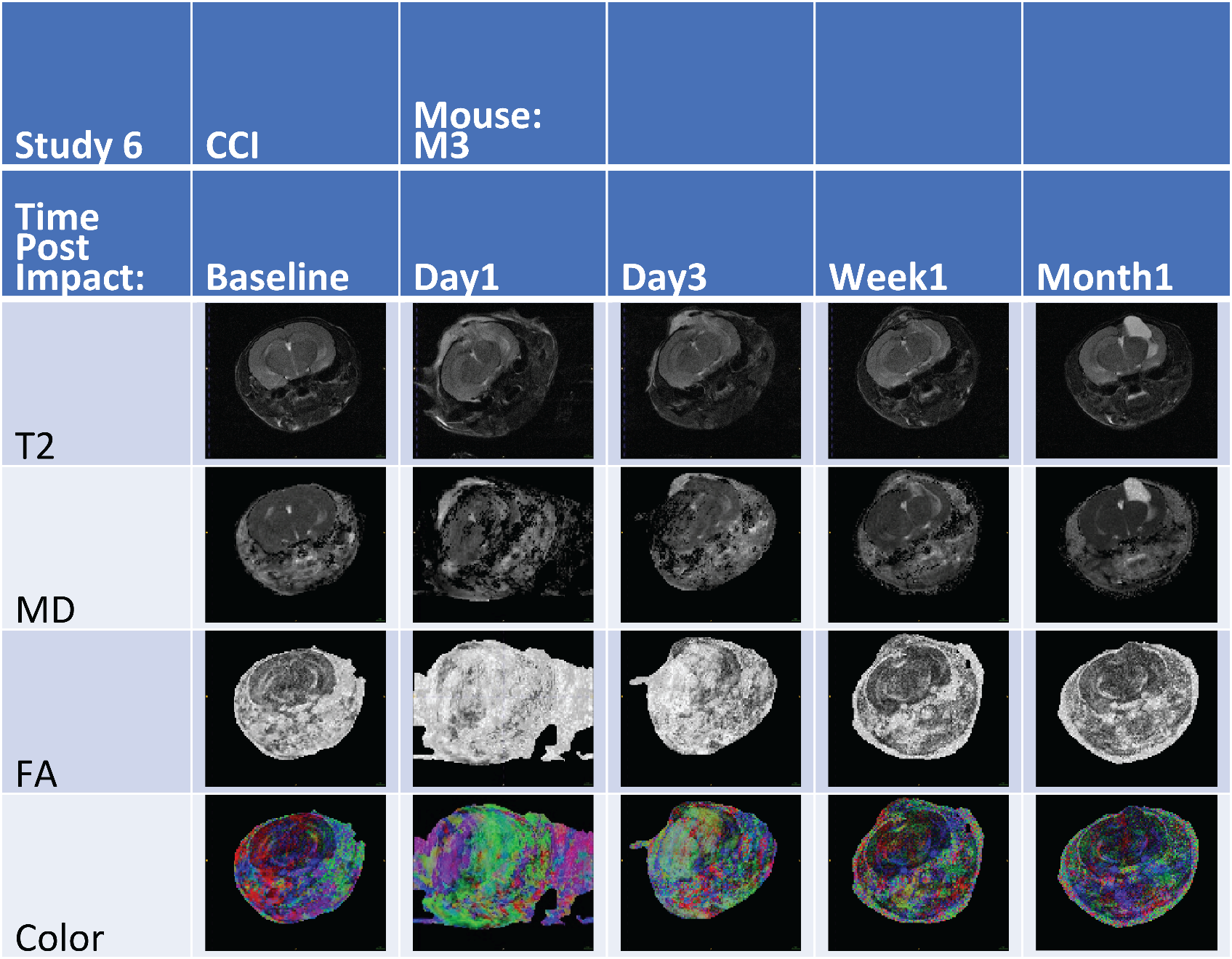


**Supplemental Figure 13**. Image summary of MRI sessions for Study6_Mouse3.
